# Epidemiologic, Genetic, Pathogenic, Metabolic, Epigenetic Aspects Involved in NASH-HCC: Current Therapeutic Strategies

**DOI:** 10.3390/cancers15010023

**Published:** 2022-12-20

**Authors:** Jorge Gutiérrez-Cuevas, Silvia Lucano-Landeros, Daniel López-Cifuentes, Arturo Santos, Juan Armendariz-Borunda

**Affiliations:** 1 Department of Molecular Biology and Genomics, Institute for Molecular Biology in Medicine and Gene Therapy, University of Guadalajara, CUCS, Guadalajara 44340, Jalisco, Mexico; 2 Tecnologico de Monterrey, EMCS, Campus Guadalajara, Zapopan 45201, Jalisco, Mexico

**Keywords:** hepatocellular carcinoma, nonalcoholic steatohepatitis, epidemiology, genetic and risk factors, pathogenesis, metabolic reprogramming, epigenetic alterations, current therapeutic strategies

## Abstract

**Simple Summary:**

Hepatocellular carcinoma (HCC) is an aggressive human cancer and is caused as consequences of chronic liver diseases. Although HCC is more common in patients with cirrhosis, there is increasing evidence that this cancer may develop in the setting of noncirrhotic nonalcoholic steatohepatitis (NASH), even simple hepatic steatosis may progress to carcinogenesis development. NASH is associated with obesity, hyperlipidemia, insulin resistance, and type 2 diabetes (T2D), which are becoming emerging risk factors for the development of HCC, as well as for cardiovascular disease. In this review, we discuss the current molecular data supporting the link between HCC and NASH, with a focus on metabolic alterations, genetic and epigenetic drivers, including current therapeutic strategies for NASH and HCC prevention and treatment.

**Abstract:**

Hepatocellular carcinoma (HCC) is the most common primary liver cancer and is the sixth most frequent cancer in the world, being the third cause of cancer-related deaths. Nonalcoholic steatohepatitis (NASH) is characterized by fatty infiltration, oxidative stress and necroinflammation of the liver, with or without fibrosis, which can progress to advanced liver fibrosis, cirrhosis and HCC. Obesity, metabolic syndrome, insulin resistance, and diabetes exacerbates the course of NASH, which elevate the risk of HCC. The growing prevalence of obesity are related with increasing incidence of NASH, which may play a growing role in HCC epidemiology worldwide. In addition, HCC initiation and progression is driven by reprogramming of metabolism, which indicates growing appreciation of metabolism in the pathogenesis of this disease. Although no specific preventive pharmacological treatments have recommended for NASH, dietary restriction and exercise are recommended. This review focuses on the molecular connections between HCC and NASH, including genetic and risk factors, highlighting the metabolic reprogramming and aberrant epigenetic alterations in the development of HCC in NASH. Current therapeutic aspects of NASH/HCC are also reviewed.

## 1. Introduction

Nonalcoholic steatohepatitis (NASH) was first described in 1980 and is the inflammatory subtype of non-alcoholic fatty liver disease (NAFLD), with steatosis as well as evidence of hepatocyte injury (ballooning and lobular inflammation), with or without fibrosis [1]. Obesity and type 2 diabetes (T2D), both pandemics in the world, probably increase the prevalence of NASH and complications such as cirrhosis and hepatocellular carcinoma (HCC) [2]. In addition, the prevalence of metabolic risk factors for NASH and HCC, including metabolic syndrome and NAFLD are increasing and may jointly become the main cause of HCC worldwide. Several comorbidities have been associated with obesity such as hypertension, which was increased in NASH-HCC, and also a higher rate of myocardial infarction and apoplectic stroke was reported among NASH-HCC patients [3]. Excessive hepatic fat accumulation in obese individuals is closely related to insulin resistance, diabetes, and dyslipidemia, thereby predisposing to the development of NASH and HCC [4]. HCC represents approximately 75–85% of primary liver cancers, being one of the most lethal and prevalent human cancers [5,6], and its pathogenesis is highly complex and heterogeneous, including genetic mutations, genetic disorders in the form of polymorphisms, epigenetic modifications, metabolic reprogramming (alterations in metabolic pathways), as well as oxidative stress, endoplasmic reticulum (ER) stress, inflammation, altered production of cytokines and adipokines, and mitochondrial dysfunction, which most of these molecular mechanisms, have been proposed in the etiology of NASH [7,8]. Oxidative stress is a critical hit in NASH pathogenesis (contributes to hepatic inflammation and fibrosis) and along with dysregulation of glucose and lipid metabolism continues throughout the entire oncogenic processes [1,9]. Furthermore, CD8^+^ T-cells and NKT-cells have been demonstrated to cooperatively promote liver damage and carcinogenesis through interaction with hepatocytes in a NASH-mouse model [10]. Other mechanisms are involved in obesity-derived hepatocarcinogenesis, including alterations in the signaling pathways of IL-6, PTEN, NF-κB, Hedgehog, TNF-α, and STAT3 [11,12]. Together, biological processes altered and these signaling pathways lead to the activation of pro-oncogenic pathways and suppression of anti-oncogenic pathways [8].

NASH and HCC are serious public health problems, and most patients are diagnosed at an advanced stage due to the complexity and diversity of drivers implicated in the development and progression of the disease. Therefore, systemic therapies are commonly recommended as the standard of medical care [6]. NASH can be partially reversed with weight loss and exercise through a combination of diet and general lifestyle changes [13]. In addition, several therapeutic drugs have been investigated with varying degrees of success, which were addressed to reduce lipid and glucose levels as well as to suppress pathological processes involved in NASH. Frontline systemic therapy of advanced HCC is constantly changing. Sorafenib, a multikinase inhibitor, was introduced in 2007 for the treatment of HCC and was the only validated treatment for many years. In 2020, the combination of atezolizumab with bevacizumab showed superiority to sorafenib alone in survival, making it the gold-standard first-line therapy. Regorafenib and lenvatinib, other multikinase inhibitors, were also approved by the United States Food and Drug Administration (FDA) in the second and first-line settings, respectively [14,15]. This review provides an update on the genetic and risk factors, as well as pathophysiology, metabolic reprogramming, and epigenetic alterations involved in the transition from NASH to HCC, and briefly summarizes current therapeutic and pharmacological strategies for the treatment of NASH and HCC derived by obesity.

## 2. Epidemiology of NASH/HCC

The epidemics of obesity and T2D are increasing risk factors for the development of NASH and HCC. Obesity is characterized as excessive fat accumulation due to excess calorie uptake and is considered, together with insulin resistance as the main contributing factor to the development of liver steatosis [1,16]. In the last decades, the prevalence of obesity has increased in the world, including in developing countries such as Brazil, Mexico, Turkey, Lebanon, and South Africa [17]. According to the World Health Organization (WHO), in 2016, 1.9 billion adults aged 18 years and older were overweight (650 million adults were obese). In 2019, an estimated 38.2 million children under the age of 5 years were overweight or obese. In addition, the number of overweight children under 5 has increased in Africa and Asia (https://www.who.int/news-room/fact-sheets/detail/obesity-and-overweight, accessed on 8 October 2022). Therefore, in the next decades, the high obesity prevalence may lead to rising numbers of NASH-associated HCC.

### 2.1. Prevalence of NASH

The prevalence of NASH in the general population is estimated to be between 1.5% and 6% [18]. In the United States, data from the National Health and Nutrition Examination Surveys (NHANES) 2017–2018, which included 4218 adults with valid elastography measurements, indicated that the prevalence of age-adjusted high-risk NASH was 5.8% and was higher among men (8.2% versus 3.6% in women) and in Hispanics (9.2% versus 5.8% non-Hispanic Asians, 5.2% in non-Hispanic whites, and 3.8% in non-Hispanic blacks). Furthermore, the prevalence of high-risk NASH among individuals with T2D is higher, ranging between 8.7% and 22.5% [19]. The high prevalence of NASH in the Hispanic population is in agreement with that published by Pomenti et al. who reported that Hispanic patients had significantly more NAFLD and alcohol-related liver disease (both *p* < 0.0001) [20]. Due to the continuing increase in diabetes and obesity, the future burden of NASH in the United States is predicted to increase considerably. According to modeling NAFLD disease burden in several countries for the period 2016–2030, NASH prevalence will increase by 15–56%, and liver mortality and advanced liver disease will more than double as a result of an aging population [21]. The prevalence of NASH in the United States will increase by 63% by 2030, while the incidence of decompensated cirrhosis will increase by 168%, and incidence of HCC will increase by 137%, and liver-related deaths by 178%; thus causing around 800,000 excess liver deaths [22]. In China population, NASH was estimated to have a prevalence between 2.4–6.1%, and the prevalence in males (6.89%) was higher than that in females (5.04%) [21,23,24,25]. A literature review found that Japanese NASH prevalence is estimated to be between 1.9 and 2.7%, and NASH is more common among males than females; however, females experience more serious diseases than males [26]. Therefore, the findings indicate that NASH prevalence is more common among Hispanics and men.

### 2.2. Prevalence of HCC

HCC is the most frequent form of liver cancer and a leading cause of cancer-linked deaths worldwide. Primary liver cancer is the sixth most commonly diagnosed cancer and the third most mortal cancer, counting with 906,000 new cases and 830,000 deaths in the world [27]. HCC accounts for between 75–85% of primary liver cancers [18], and it has tripled in the last few decades in high-income countries (1.4 per 100,000 subjects per year in 1975–1977 to 4.8 per 100,000 in 2005–2008), an increasing incidence associated mainly to the increasing prevalence of obesity and NAFLD/NASH [1,28]. Most HCC cases occur between 30 and 60 years of age [29]. HCC predominates in Asia with 72% of cases (more than 50% in China), 10% in Europe, 7.8% in Africa, 5.1% in North America, 4.6% in Latin America, and 0.5% in Oceania [30]. However, it was reported that American Indians/Alaska Natives has the highest incidence (11.4), followed by Hispanics (9.8) and Asians/Pacific Islanders (9.1) in 2018. In addition, black individuals have lower odds of early-stage HCC and the worst survival among different populations in the United States, while Asian and Hispanic individuals have better survival [31]. Moreover, the estimated global incidence rate of liver cancer per 100,000 person-years was 9.3, and the mortality rate was 8.5 in 2018 [32]. Eastern Asia and sub-Saharan Africa regions have the highest incidence rate of HCC, meanwhile, in the United States, HCC incidence rates have tripled over the last three decades [33]. In fact, approximately 72% of global liver cancer cases are reported in the Asian population in 2020 [31]. With respect to this, significant disparities in HCC were observed in recent years in different race/ethnic populations in the United States, where Hispanics have a higher incidence (approximately 36%) and mortality. Hispanic patients showed a median overall survival of 1.4 years (95% confidence interval [CI], 1.22–1.56) which was similar to that of non-Hispanic White patients (1.3 years; 95% CI, 1.26–1.41; *p* = 0.07). Furthermore, Hispanic patients showed larger tumors, more advanced-stage disease, and elevated rates of macrovascular invasion and extrahepatic spread, including more likely to have metabolic risk factors for chronic liver disease and obesity [20,34]. For men, the incidence rate increases until 45 years, while for women up to 60 years [33]. HCC is more frequent in males than in females (with a ratio of 2.4:1), and it has an average five-year survival of <15% [35]. However, the reason for gender disparity in the incidence proportion of HCC is not well understood. Differences between steroid sex hormones, epigenetic modifications, and immune response may contribute to higher rates in men [33]. HCC is infrequently diagnosed before the age of 40–50 years, and the age distribution of HCC is different between regions and countries. For instance, in Europe and North America is between 63–65 years, while in China the mean age interval at diagnosis is 55–59 years, and in low-risk populations, 75 years or older [33]. HCC is one of the rapidly growing causes of mortality in cancer patients, which has been increasing in North America and several European regions and decreasing in traditionally high-risk regions, such as Japan and parts of China [36]. This trend may be caused by risk factors such as metabolic syndrome, NAFLD, population genetics, excessive alcohol consumption, obesity, diabetes, and different environmental exposures. Hispanic patients have a 5-fold greater risk of HCC mortality based on the Third NHANES 1988–1994, with Hispanic ethnicity being an independent predictor of mortality (HR 5.14; 95% CI, 1.75–15.06) [34]. It is predicted that the global burden of HCC will increase to more than 22 million cases over the next two decades [37]. Further epidemiological and transnational studies are needed for a better understanding of ethnic susceptibility and hepatic tumor prevention.

### 2.3. Incidence of HCC in Patients with NASH

Few studies have been published on NASH-associated HCC prevalence. Most authors agree that the main risk factors of transition from NASH to HCC are liver fibrosis, diabetes, insulin resistance, obesity, age, and male gender; and the concurrent NASH is becoming a dominant factor of hepatic cirrhosis and HCC [38,39]. HCC incidence is very rare in patients with simple steatosis or hepatic steatosis without necroinflammation or fibrosis. However, in patients with NAFLD-related cirrhosis, the incidence of HCC is high [40]. NAFLD is already the fastest-growing cause of HCC in the United States, France, and the UK. Recently, the estimated annual incidence of HCC ranges from 0.5% to 2.6% among patients with NASH cirrhosis [41]. Although subjects with NASH appear to have a lower risk of HCC than subjects with HCV-associated cirrhosis, the annual incidence is around 1–2%. In addition, it was reported in a large cohort study of 4235 patients with NASH cirrhosis from the Veterans Health Administration in the United States, the incidence of HCC was calculated to be 1.06 per 100 person-years [30]. Several authors have reported an increase in occurrences of HCC in the course of NAFLD. For example, in the UK, in the years 2000–2010, the rate of cancers associated with NASH increased approximately from 21% to 35%. In the United States between 2004 and 2009, the annual growth of NASH-related HCC was 9%. In Asia, the rate of nonviral cases of HCC in the years 1991–2010 increased approximately from 10% to 24%. An increase was also reported in the HCC percentage, reaching 37% of cases, which emerged in the course of NASH without concomitant hepatic cirrhosis [39,42]. In a German single-center study, the percentage of NASH-associated HCC was 24%, while in one study from Turkey the percentage of NASH-associated HCC was 3.5% in 2010 [17]. Another study from the United States reported that the prevalence of NASH-related HCC increased by 68% from 2010 to 2015 [43]. In addition, a cross-sectional study from the United States included 218,950 patients with NASH and 30,493,574 patients without NASH, and found that HCC prevalence in subjects with NASH was 0.50% [95% CI, 0.41–0.59] compared to 0.21% (95% CI, 0.20–0.23) in subjects without NASH (*p* < 0.001), and NASH patients are 60% more likely to develop HCC compared with patients without NASH [44]. In 2018 was estimated the trends in the prevalence of HCC in 158,347 adult liver transplant candidates. Of these, 26,121 (16.5%) had HCC, and 2690 patients (11%) were identified as having NASH as the cause of HCC [45]. Information regarding the distribution of NASH-related HCC cases is not easily available, particularly several factors involved in HCC development that may complicate this issue and could lead to erroneous numbers.

## 3. Risk Factors for NASH and HCC

### 3.1. Obesity Is Associated with NASH and HCC

Excess body weight was found to be associated with an increased risk of liver cancer in a meta-analysis of 11 cohort studies. Compared with persons of normal weight, the summary relative risks of liver cancer were 1.17 (95% CI: 1.02–1.34) for overweight patients and 1.89 (95% CI: 1.51–2.36) for obese patients [46]. According to another meta-analysis, which included 26 prospective cohort studies with a total of 25,337 primary liver cancer cases, summary relative risks (SRRs) with their corresponding 95% confidence intervals (CIs) were calculated using a random-effects model. Excess body weight [EBW: body mass index (BMI) ≥ 25 kg/m^2^] and obesity (BMI ≥ 30 kg/m^2^) were associated with an elevated risk of primary liver cancer, with significant heterogeneity (EBW: SRRs 1.48, 95% CIs 1.31–1.67, *p*(h) < 0.001, I2 = 83.6%; Obesity: SRRs 1.83, 95% CIs 1.59–2.11, *p*(h) < 0.001, I2 = 75.0%). Furthermore, Obese males had a higher risk of primary liver cancer than obese females (*p* = 0.027) [47]. In addition, patients with HCC related to NASH had higher BMI (27 kg/m^2^) than patients with HCC with induced HCV (24 kg/m^2^). With a BMI greater than 30 kg/m^2^ the risk of HCC almost doubles, and with BMI greater than 35 kg/m^2^ it elevates almost 4-fold [39]. Notably, obesity has been identified in early adulthood as a significant risk factor for HCC, with odds ratios of 2.6 (95% CI: 1.4–4.4), 2.3 (95% CI, 1.2–4.4), and 3.6 (95% CI, 1.5–8.9) for the entire population, for men, and for women, respectively [48]. In addition, one meta-analysis of nine studies with more than 1.5 million individuals with obesity found that these patients had a 2-fold increased risk of HCC-related mortality [49].

### 3.2. Metabolic Syndrome Is a Risk Factor for NASH and HCC

Metabolic syndrome includes conditions such as abdominal obesity, hypertriglyceridemia, low HDL-cholesterol, hypertension, and hyperglycemia (insulin resistance), which is involved in the development of both benign and malignant hepatic diseases [50]. Moreover, certain health status, such as obesity, hypertension, and diabetes, increases the risk of NASH [1]. A meta-analysis has shown a 1.76-fold risk of HCC development and a 2.41-fold risk of liver-related death in individuals with metabolic syndrome [51]. Population attributable fraction (PAF) is the part of cases with disease (i.e., HCC) that can be avoided by deleting the underlying risk factor (i.e., NAFLD), and PAF is calculated through prevalence (how common) and risk estimate (how strong). It was reported that metabolic disorders (i.e., obesity, diabetes, metabolic syndrome, and NAFLD) carry the greatest HCC PAF risk, followed by other risk factors. Furthermore, metabolic disorders carried the greatest HCC PAF risk among Hispanics (PAF 39.3%; 95% CI 31.9–46.7%), followed by non-Hispanic whites (PAF 34.8%; 95% CI 33.1–36.5%), Asians (PAF 21.8; 95% CI 16.5–27.1%), and African Americans (PAF 14.4%; 95% CI 6.4–22.3%) [52]. 

### 3.3. Diabetes Increase the Risk of NASH and HCC

There is a relationship between NASH and T2D. The prevalence of T2D in patients with NASH was reported to be approximately 36%, and T2D increases the risk of severe NASH and advanced fibrosis [53,54]. T2D is an independent factor for HCC, and is associated with a 2- to 3-fold increase in the risk of HCC [55], and death risk due to HCC in the course of diabetes rises by 1.56 [39]. A systematic review and meta-analysis involving 17 case-control studies and 32 cohort studies showed a significantly increased risk of HCC prevalence among diabetic patients (RR = 2.31, 95% CI: 1.87–2.84). Also meta-analysis of 7 cohort studies found an increased risk of HCC mortality (RR = 2.43, 95% CI: 1.66–3.55) for individuals with diabetes [56]. In addition, T2D is more strongly associated with elevated HCC risk in individuals with NASH-related cirrhosis than in cirrhosis of other etiologies [40]. Additionally, the presence of metabolic syndrome together with T2D increases the risk of HCC 5-fold [18].

### 3.4. NAFLD Is the Main Risk Factor of NASH and HCC

**Figure 1:**
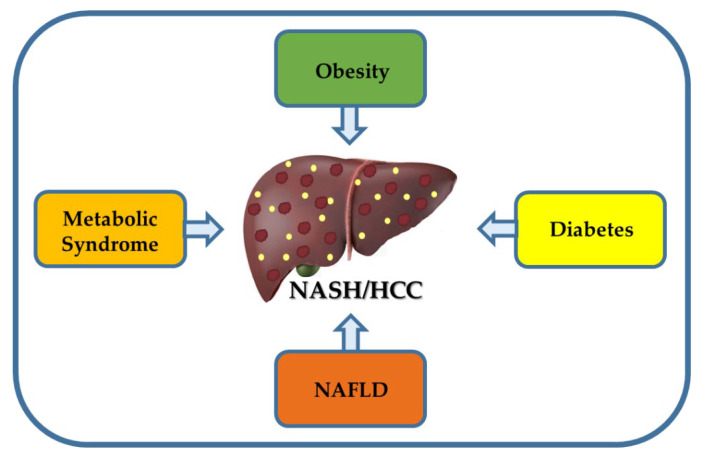
Main risk factors for NASH and HCC development. Obesity is associated with the development of metabolic syndrome, diabetes, NAFLD, NASH, and HCC, which may act synergistically to accelerate the onset and development of NASH-derived hepatocarcinogenesis.

The prevalence of NAFLD is increasing in line with obesity and 90% of the morbidly obese have NAFLD. It is estimated that 25% to 30% of the adult population is thought to be living with NAFLD [18]. In addition, NAFLD has become the leading cause of chronic liver disease in most regions of the world, especially in developed and many industrialized countries, including the United States (30% prevalence) [36]. Approximately 20–30% of patients with NAFLD progress to NASH, and up to 40% of NASH patients can develop fibrosis, while 10–20% of NASH patients may progress to cirrhosis [31,57,58]. The association of HCC with NAFLD has been well described, and NAFLD is considered the most frequent underlying HCC risk factor (59%) followed by T2D (36%) and HCV infection (22%) [17,18]. It is important to note that cirrhosis increases the risk of HCC in patients with NASH, as it does in patients with other comorbidities [18]. As already mentioned, several risk factors for NAFLD are also independently associated with HCC (i.e., obesity, diabetes, and Hispanic ethnicity, among others), which may explain the association between NAFLD and HCC, especially in the absence of fibrosis or cirrhosis. In fact, about 20% to 50% of NASH patients without cirrhosis may develop HCC [40]. However, the contribution of NAFLD without cirrhosis to the burden of HCC has not been clearly defined [59]. Additionally, patients with NAFLD fibrosis stages 3 and 4 have approximately a 7-fold higher risk for developing HCC than those without hepatic disease [59]. Therefore, there exists several risk factors for HCC in patients with NAFLD such as the presence of diabetes (insulin resistance), obesity, older age, and male sex [18]. In summary, the main obesity-associated risk factors for NASH and HCC are illustrated in Figure 1.

## 4. Genetic Factors and Gene Expression Affecting Development of NASH and HCC 

### 4.1. Genetic Risk Factors for NASH

Obesity and insulin resistance are considered the major contributing factors to the development of hepatic steatosis. However, the heterogeneous prevalence of hepatic steatosis between races/ethnicities suggests other influencing factors, such as genetic variance [16]. A study by Younossi et al. found by multivariate analysis that NASH was independently associated with being Hispanic (OR, 1.72; 95% CI, 1.28–2.33) and less associated with being African-American (OR, 0.52; 95% CI, 0.34–0.78). Furthermore, Hispanics have common components of metabolic syndromes, such as hypertension (*p* < 0.05) [60]. Genetic polymorphism, such as variations of single nucleotide polymorphisms (SNPs) may influence not only hepatic fat accumulation, but also contribute to a more aggressive disease course. The patatin-like phospholipase domain-containing protein 3 (PNPLA3) has hydrolase activity towards triglycerides and retinyl esters, and promotes lipid droplet remodeling in hepatocytes and hepatic stellate cells (HSCs) [61,62]. The *PNPLA3* gene polymorphism is associated with NASH. Romeo et al. first demonstrated that the rs738409 [G] allele in the *PNPLA3* gene was significantly associated with elevated hepatic fat levels (*p* = 5.9 × 10^−10^) and liver inflammation (*p* = 3.7 × 10^−4^). In addition, the allele was more commonly seen among Hispanics (0.49) compared to non-Hispanic whites (0.23) and African Americans (0.17) [63]. Similarly, Wagenknecht et al. demonstrated that PNPLA3 rs738409 [G] was two times more common in Hispanics compared to African Americans (40% versus 19%), which is consistent with more prevalence of NAFLD among Hispanics compared to African Americans [64]. Furthermore, G allele homozygotes have greater than a 2-fold increase of liver fat content compared to noncarriers [63]. A meta-analysis of 16 studies (2124 subjects with NAFLD) concluded that GG homozygotes have not only 73% higher hepatic fat content, but also more than 3-fold higher risk of NASH, 3.24-fold greater risk for necroinflammatory scores and 3.2-fold greater risk of developing fibrosis compared to the CC carriers (*p* < 1.0 × 10^−9^) [65]. PNPLA3 rs738409 GG has also been confirmed as heredity risk factors associated with NASH in the Chinese population [66,67]. In the PNPLA3 I148M (rs738409) polymorphism, the isoleucine (I) is changed to methionine (M) at position 148 of the protein, and the PNPLA3 I148M mutant protein exhibits reduced enzymatic activity [61]. In line with this, mice fed the NASH-inducing diet and GalPLAC3-conjugated antisense oligonucleotide-mediated PNPLA3 silencing decreased liver steatosis. Meanwhile, I148M/M knock-in mice for PNPLA3 also lower inflammation and hepatic fibrosis degree [68]. Therefore, Pnpla3 silencing exerts a beneficial effect on NASH. 

Other SNPs are peroxisome proliferator-activated receptor gamma coactivator-1-alpha (PPARGC1A) rs8192678 GA/AA genotypes and transmembrane 6 superfamily member 2 (TM6SF2) rs58542926 T allele, which have been confirmed as heredity risk factors associated with NASH in Asia population [69,70]. PPARGC1A rs8192678 risk A allele was also independently associated with NAFLD and NASH patients [71]. The TM6SF2 rs5854026 C/T polymorphism results in the replacement of glutamate with a lysine in the residue 167 (E167K), and this variant is susceptible to NASH progressive forms due to a higher degree of steatosis, inflammation, and fibrosis, but is protected against cardiovascular disease [72]. The haptoglobin (Hp) 2-2 genotype also appeared to contribute to NASH development in the Asian population [73]. On the other hand, Namikawa et al. reported that Japanese patients with NASH had a higher incidence of the rs4880 T/T superoxide dismutase 2 (SOD2) genotype [74]. Other SNPs have a role in the protection against NASH development. For example, the 17b-Hydroxysteroid dehydrogenase 13 (HSD17B13) rs72613567 variant leads to the synthesis of a truncated loss-of-function enzyme, and it was associated with a reduced risk of NASH and progressive liver damage (protects against ballooning degeneration, lobular inflammation, and fibrosis) [75,76]. Another protective SNP is uncoupling protein 2 (UCP2) rs695366, which elevates its expression and decreases the risk of NASH progression [77]. Therefore, the *PNPLA3* I148M and *TM6SF2* E167K gene variants are major determinants for interindividual differences in liver steatosis and susceptibility to NASH.

### 4.2. Genetic Risk Factors for HCC

Ethnicity has been associated with the risk of HCC. Genetic polymorphisms in several genes have been associated with an increased risk of HCC, including variants in *PNPLA3, TM6SF2, GCKR,* and *MBOAT*; meanwhile, *HSD17B13* variants decreased the risk of HCC [78,79,80]. However, Gellert-Kristensen et al. reported that a genetic risk score comprising three common variants in PNPLA3, TM6SF2, and HSD17B13 (rs72613567) was associated with up to 12-fold higher risk of cirrhosis and up to 29-fold higher risk of HCC [80]. In addition, for each of the three variants, subjects carrying two risk alleles have a 2- to 4-fold higher risk of developing fibrosis, cirrhosis, and HCC compared to those without these risk alleles [78,81,82]. Another PNPLA3 rs738409 [G] risk allele is observed in 40% of the European population, and increases the risk of HCC development by 12-fold. Furthermore, GG homozygosity is associated with the development of HCC at a younger age, diffuse-type HCC, and poor prognosis [83]. Moreover, PNPLA3 rs738409 polymorphism is an independent risk factor for HCC among patients with NASH or alcohol-related cirrhosis [84]. A systematic review and meta-analysis analyzed the human hereditary hemochromatosis C282Y and H63D polymorphisms, and this study found that C282Y was associated with increased HCC susceptibility in the overall population, while H63D increased the odds of developing non-cirrhotic HCC in the African population [85]. Donati et al. published that the MBOAT7 rs641738 T allele is associated with reduced MBOAT7 expression and may predispose to HCC in patients without cirrhosis [86]. These findings indicate that genetic predisposition plays an important role in the risk of developing HCC. Table 1 summarizes the genetic variants implicated in NASH and HCC, and other liver damage. 

### 4.3. Genetic Instability and Altered Gene Expression Affecting NASH and HCC Diseases

#### 4.3.1. Somatic Mutations in NASH and HCC [77,88]

Genetic predisposition and gene expression represent a significant risk regarding disease progression to NASH and HCC. Next-generation sequencing provides a better understanding of genetic factors related to the initiation and progression of hepatic cancer in recent years. *TM6SF2* mutations are frequently found in patients with NASH [92]. In addition, the presence of the hemochromatosis C282Y mutation was a risk factor for the development of advanced hepatic fibrosis among United States Caucasian patients with NASH [93]. Desterke et al. reported that 25 genes are involved in NASH development and 44 genes in the advancement from NASH to HCC. Furthermore, of the 25 genes found for NASH, 22 were upregulated and three were downregulated, when compared with healthy obese patients without NASH [94]. The *YWHAZ* gene plays an important role in driving HCC tumor progression according to the analysis results [94,95]. 

The most frequently mutated genes in HCC cases were reported by the Cancer Genome Atlas (TCGA) Research Network, including the *TERT* promoter, *TP53,* and *CTNNB1* [96]; and along with low-frequency mutated genes (e.g., *AXIN1, ARID1A, ARID2 and RB1, BAP1, NFE2L2, KEAP1, TSC1/TSC2, MLL2*, *ALB* (Albumin), and *APOB* mutations), help define some of the core deregulated pathways in HCC [97]. In line with this, NASH-HCC samples revealed that *TERT* promoter (56%), *CTNNB1* (28%), *TP53* (18%), and *ACVR2A* (10%) were the most frequently mutated genes [98]. Interestingly, Asian Americans showed a higher mutational frequency in *TP53, RB1,* and higher VEGF-binding pathway compared with European Americans, while *IL-17* was mutated only in European American population. Additionally, three putative oncogenic genes, including *TRPM3, SAGE1,* and *ADAMTS7*, were mutated exclusively in the Asian population [99]. It was reported that patients with HCC have genetic alterations (e.g., mutations, fusion, deletions, amplifications, or single nucleotide polymorphisms) in several genes, and genes previously identified as key players in the NASH progression and also implicated in liver cancer, showed genetic alterations, especially mutations in five genes (*DGAT1, FASN, LPL, IRS2*, and *YWHAZ*), which are implicated in lipid synthesis, insulin resistance, and cancer progression [94]; thus, these genomic alterations are contributing in the progression of the disease from NASH to HCC. 

Several mutated genes were identified in a murine NASH-associated HCC model, which has also been reported as drivers in other human cancers, including *Mtor, Sdk1, Braf, Pik3cb, Ctnnd1, Ctnna3, Akt3*, and *Nos* [100]. Liang et al. identified 82 genes to be recurrently mutated in two or more NASH-HCCs; which included two genes mutated in 4/5 NASH-HCCs (*Ryr1* and *Sdk1*), 8 genes mutated in 3/5 NASH-HCCs (*Epha8, Pcdh15, Fat2, Cep152, Ttn, Rxfp1, Aox3l1,* and *Pkd1l3*), and 71 genes mutated in 2/5 NASH-HCCs (notably *Mtor, Ryr2, Cacna1h, Col7a1, Fcgbp, Adam29, Gpr98,* and *Pclo*) [100]. These findings suggest that mutations of these cancer-driver genes contribute to the development of cholesterol-associated NASH-HCC, and the authors suggest that *Mtor* and *Sdk1* may also function as cancer drivers in NASH-HCC [100]. 

#### 4.3.2. Pathways Affected by Gene Mutations in a Mice NASH-HCC Model and Human NASH-HCCs

To investigate the signaling networks with mutations in NASH-HCCs, Liang et al. conducted KEGG pathway analysis; and they found seven pathways significantly enriched by gene mutations in three or more NASH-HCCs, which include metabolic, insulin and calcium signaling, cell adhesion and tight junction molecules, ABC transporters, and axon guidance [100]. In addition, gene mutations expected to dysregulate calcium signaling were found in all five dietary cholesterol-associated NASH-HCCs [100], as calcium signaling is affected by cellular cholesterol content and plays an important role in cancer development [101,102]. Of note, twenty-eight calcium signaling genes were found mutated in NASH-HCCs, with 6 frequently mutated (*Ryr1, Ryr2, Cacna1d, Cacna1h, P2rx1,* and *Itpr1*) and all encoding calcium channel proteins [100]. Furthermore, 16 mutated genes are involved in insulin signaling, such as insulin receptor (*Insr*), and some other well-known cancer-related genes (*Mtor, Hras1, Akt3,* among others), with *Mtor* and *Hras1* commonly mutated. Other 75 mutated genes were expected to dysregulate 15 specific metabolic signaling pathways, including 6 associated with lipid metabolism [100]. Another study reported that an androgen receptor (AR)-driven oncogene, cell cycle-related kinase (CCRK), cooperates with obesity-induced pro-inflammatory signaling to promote NASH-related hepatocarcinogenesis. Moreover, activation of the mTORC1/4E-BP1/S6K/SREBP1 cascades via GSK3 phosphorylation and the components of the STAT3-AR-CCRK-mTORC1 pathway are concordantly overexpressed in human NASH-associated HCCs [103]. 

In 37 human NASH-HCCs, 21 genes were recurrently mutated in at least two human NASH-HCCs. These included *MTOR* (insulin signaling), *SDK1* (cell adhesion molecule), and three calcium signaling genes (*RYR1, RYR2,* and *CACNA1H*) [100]. Moreover, in human NASH-HCCs, most of the genes mutated (11/19) encoded calcium channels proteins (*RYR1, RYR2, CACNA1B, CACNA1E, CACNA1H, CACNA1I, GRIN2C, ATP2A2, ATP2B4, SLC8A1*, and *ITPR3*). The nine *RYR1* mutations identified in both animal models and human NASH-HCCs were located across the whole gene, with 3 stop gains or truncating mutations inferring likely loss-of-function mutations [100]. Therefore, genetic disruptions of calcium channels and calcium homeostasis, including other gene mutations may be important to the contribution of NASH-related carcinogenesis. 

#### 4.3.3. Dysregulated Gene Expression in NASH and HCC

Development and progression of NASH and HCC is a multistep event, which involves dysregulated gene expression implicated in metabolism signaling, inflammation, oxidative stress, and several signaling pathways involved in hepatocarcinogenesis, including dysregulation of proliferation, differentiation, and neoangiogenesis. Several upregulated genes have been found in the progression of NASH, for example, levels of nicotinamide adenine dinucleotide phosphate reduced (NADPH) oxidase 4 (NOX4) were found to increase in patients with NASH compared with controls [104]. NOXs are a key producer of reactive oxidative species (ROS) in liver cells. Hepatocyte-specific deletion of *NOX4* reduced oxidative stress, lipid peroxidation, and liver fibrosis in mice with diet-induced steatohepatitis, and mice treated with GKT13783 increased insulin sensitivity [104]. Brahma-related gene 1 (*Brg1*), a chromatin remodeling protein, is upregulated in NASH and primary hepatocytes exposed to free fatty acids. Furthermore, Brg1 contributes to the transcription of pro-inflammatory mediators possibly by regulating the interaction between NF-κB and its co-factor MRTF-A; and liver injury and hepatic inflammation are attenuated in hepatocyte-specific *Brg1* knockout mice fed with a methionine- and choline-deficient diet (MCD) [105]. In liver samples from patients with NASH is also upregulated the expression of X-box binding protein-1 (XBP1) [106], which regulates the mammalian unfolded protein response (UPR) or ER stress response [107]. Hepatocyte-specific Xbp1 deficiency inhibited the development of NASH in mice diets, and *Xbp1*-deleted macrophages reduced steatohepatitis by decreasing the expression of nucleotide-binding domain leucine-rich repeat-containing receptor (NLR) family pyrin domain containing 3 (NLRP3) and secretion of pro-inflammatory cytokines [106]. Desterke and Chiappini identified a signature of 25 genes that are commonly dysregulated during steatosis progression to NASH and cancer. Expression heatmap revealed that 22 of 25 lipid-related genes were overexpressed in NASH liver samples, and three were downregulated (*PPARA, PPARGC1A,* and *CNPB*) [94]. The genes upregulated in NASH are implicated in cholesterol storage (*LPL, CD36*, and *SREBF2*), long-chain fatty acid import (transporters *SLC27A4* and *CD36*), and triglyceride biosynthesis (*FASN, DGAT1*, and *LPL*) [94]. The expression of genes involved in Wnt signaling is controversial in NASH, Wnt signaling may be downregulated in NASH [108], or the expression of Wnt signaling genes may be upregulated in NASH compared to steatosis, including *Ctnnb1* and *Myc* [100]. The controversial results of *Wnt* gene expression in NASH could be related to the genetic background (e.g., somatic mutations, SNPs, epigenetic modifications, among others), comorbidities, and other factors implicated in NASH pathogenesis.

Other genes upregulated were *BCL2A1*, an anti-apoptotic player, usually implicated in cancer progression, as well as *YWHAZ, YWHAH*, and *IRS2*, which participates in the development of insulin resistance as well as hepatocellular carcinoma. In addition, six genes are involved in cancer and inflammatory processes (*YWHAZ, CCL2,* and *SMPD2*) and lipid droplet formation and metabolism (*CIDEC, VLDLR,* and *FASN*) and were significantly increased in NASH patients [94]. In addition, Desterke and Chiappini identified genes implicated in the progression of NASH to HCC, which include genes involved in the regulation of fatty acid and cholesterol metabolisms such as *LPL, VLDLR, LIPA, ANXA2*, and *PLEK*; lipid accumulation (*CIDEC, PLIN1*); and metabolism (*PPARA* and *BCL2A1*). Additionally, five genes were associated with poor prognoses such as *FASN, DGAT1, LPL, IRS2*, and *YWHAZ*. Four of these genes are implicated in lipid metabolism regulation and one in liver cancer [94]. Another study using a combined analysis of an assay for transposase-accessible chromatin with high throughput sequencing (ATAC-seq) and global gene expression data in NASH-derived HCC tissue samples, identified 139 upregulated and 60 downregulated genes. Interestingly, 15 of the 139 upregulated genes had accessible chromatin sites within 5 Kb of the transcription start site, including *Apoa4, Anxa2, Serpine1, Igfbp1*, and *Tubb2a*, which are involved in the development of NASH and HCC [109]. *Apoa4* is involved in the regulation of multiple metabolic pathways such as lipid absorption, lipid metabolism, and glucose homeostasis, and the overexpression of APOA4 promotes lipid accumulation in the liver [110]. The *Anxa2* gene encodes calcium-dependent phospholipid-binding protein ANXA2, and is a key contributor to several hallmarks of cancer, including cancer cell proliferation, inhibition of apoptosis, cell adhesion, invasion, metastasis, and angiogenesis. Several studies have shown an upregulation of ANXA2 in human hepatocarcinogenesis [111,112]. There are several genes and signaling pathways, including metabolic pathways involved during NASH progression to HCC.

#### 4.3.4. Circulating miRNA Signature Associated with NASH and NASH-HCC

Non-coding RNAs (ncRNAs) are groups of RNAs with little or no protein-coding potency which can affect diverse cellular functions and participate in pathological processes. Based on length, ncRNAs are classified into micro RNAs (miRNAs, 18–24 nt), long noncoding RNAs (IncRNAs, > 200 nt), small noncoding RNAs (ncRNAs, <200 nt), and recently emerged circular RNA (circRNA), noncoding RNAs formed with a covalently closed loop. 

Micro RNAs are involved in the regulation of gene expression by suppressing or degrading their translation; and regulate a wide spectrum of biological processes, including lipid synthesis, fatty acids and glucose catabolism, inflammation, cell differentiation, proliferation, apoptosis, and necrosis, which have been known to be epigenetically deregulated in NAFLD, NASH, and HCC [113]. MiR-122 is the most abundant miRNA in the human liver, and has been proposed as a marker of NASH and disease severity [113]. However, several reports showed an opposite association in the liver, with hepatic miR-122 expression downregulated in NAFLD and NASH, both in animal models and human patients [114,115]. In genetically improved farmed tilapia (GIFT, Oreochromis niloticus), was demonstrated that inhibition of hepatic miR-122 led to upregulation of lipogenic genes, among which stearoyl-CoA desaturase gene (SCD) at the 3’-UTR region, thus affecting lipid metabolism and contributing to fat accumulation [116]. In contrast, Long et al. found that free fatty acids (FFAs) or high-fat diet (HFD) increased miR-122 in hepatocytes-derived HepG2 and HuH7 cell lines, and in mice, respectively, revealing that miR-122 may stimulate lipogenesis by inhibiting Sirt1. Furthermore, miR-122 knockdown mitigated hepatic steatosis via the Sirt1-induced liver kinase B1/AMP-activated protein kinase (LKB1/AMPK) pathway [117]. MiR-29a has been found to reduce liver inflammation and fibrosis following a liver injury. It was demonstrated that increased miR-29a function inhibits DNA methyltransferases signaling, thus hindering HSCs activation [118]. MCD-miR29a transgenic mice resulted in the downregulation of DNMT3b, TGF-β, IL-6, heme oxygenase-1 (HO-1), p-SMAD3, PI3K, and L3BII expression within the liver tissue [119]. In addition, overexpression of miR-29a in SMMC-7721 cells, a steatosis hepatic cell model, significantly decrease the 3-hydroxy-3-methylglutaryl coenzyme A reductase (HMGCR) and free cholesterol accumulation in the cells [120]. Several studies also have shown that miR-33 family members are important regulators of lipid metabolism and transport, and their expressions were increased in NASH patients [115]. Liver miR-21 is overexpressed in patients with NASH, and miR-21 inhibition or suppression decreases hepatic injury, inflammation, and fibrosis, by restoring PPARα expression [121]. An increase in miR-34a-5p and reduce in miR-122-5p and miR-29c-3p in patients with NASH were observed (*p* < 0.05). Moreover, significant correlations between miR-122-5p and unfavorable lipid profiles, including hs-CRP and miR-34a-5p were also reported [122]. MiR-34a is also upregulated in serum samples of NAFLD/NASH patients, and miR-34a downregulates the expression of several key genes in NAFLD pathogenesis, including *HNF4α, PPARA,* and *SIRT1* expressions, which are associated with higher triglycerides accumulation and liver steatosis [115]. In addition, miR-34a inhibition prevents lipid accumulation in the liver and might be used in NASH patients due to its role in regulating oxidative stress and inflammation [123]. Another miRNA involved in NAFLD is miR-21, which was upregulated in the serum of NASH patients, and its downregulation resulted in reduced hepatic inflammation [115]. Overexpression of miR-221/222 causes liver fibrosis. In addition, miR-221 has been suggested as a noninvasive serum biomarker for liver fibrosis and cirrhosis [124]. In male inbred C57BL/6J and DBA/2J mice fed with a lipogenic methyl-deficient diet, which causes liver injury similar to human NASH, were detected an increased expression of miR-34a, miR-155, and miR-200b. The livers of C57BL/6J mice were characterized by pronounced downregulation of miR-29c, whereas its expression in the livers of DBA/2J mice did not change [125]. Another group observed overexpression of the miR-34a and miR-146b in the liver of NASH subjects, which correlated with steatohepatitis development [114]. In visceral adipose tissue samples from NASH patients, the expression of miR99b and miR-197 were associated with pericellular fibrosis [126].

Pirola et al. also reported that miR-122, miR-192, miR-19a, miR-19b, miR-125b, and miR-375 were upregulated > 2-fold (*p* < 0.05) either in simple steatosis or NASH. Indeed, miR-19a/b and miR-125b were correlated with biomarkers of atherosclerosis [127,128]. It was also confirmed that serum levels of miR-192, miR-34a, and miR-22 elevated and miR-197 reduced in NASH patients [128,129]. A study reported 21 miRNAs expressed in patients with NASH, in which the expression levels of miR-23a3p, miR-224-5p, miR-26b-5p, miR-15b-5p, miR-26a-5p, miR374a-5p, miR-200c-3p, miR-1-3p, miR-let-7a-5p, miR-21-5p, let7c-5p, miR-143-3p, miR-423-5p, miR-155-5p were increased (*p* < 0.05), and the expression levels of miR- 195-5p, miR-130b-3p, miR-16-5p, miR-19a-3p, miR-19b-3p, miR-27a-3p, miR-93-5p were suppressed (*p* < 0.05) in the group of patients with NASH [130]. Authors suggest that probably the genes responsible for adenosine triphosphate (ATP) production were suppressed by increased miRNAs (miR-let-7c-5p, miR-155-5p, miR-423-5p, miR-15b-5p, miR143-3p, and miR-26a-5p) in the group of patients with NASH compared to the steatosis group [130]. In addition, miR-Let-7a-5p, miR-let-7c-5p, miR-15b-5p, miR-26a-5p, miR-423-5p, inhibit the AMPK complex, altering the energy balance in the liver, contributing to the development of NASH. The increase in miR-423-5p suppresses the expression of the *PPARA* gene, which may induce a decrease in fatty acid oxidation and an increase in lipogenesis in the liver [130]. On the other hand, the reduction of the activity of IKB, RelA, and JUN by miR-423-5p may lead to inhibition of the NF-κB pathway, including inactivation of pro-apoptotic factors, thus contributing to the progression of NASH [130]. MiR-let-7c-5p upregulates the *IL1B* gene in NASH patients, and it has been shown that an increase in IL-1β production leads to the activation of fibrogenic responses in HSCs, including inflammation and secretion of tissue inhibitor of metalloproteinase-1 [131]. Therefore, suppression of IL-1β expression by increasing miR-let-7c-5p may lead to a reduction in NASH progression. IL-6R is decreased by upregulated miR-423-5p, miR-let-7a-5p, and miR-let-7c-5p in patients with NASH [131]. IL-6R was identified as a direct target of miR-let-7a, and the miR-let-7a inhibitor significantly increased the level of IL-6R expression, resulting in increased proliferation, reduced apoptosis, and inhibited inflammatory response in ATDC5 cells, which are derived from mouse teratocarcinoma [132]. The increase of miR-let-7c-5p is predicted to target the *VCAM1* gene, and pharmacological inhibition or genetic deletion of *VCAM1* in liver endothelial cells reduced liver inflammation, injury, and fibrosis in mice with NASH [133]. Additionally, an increase in miR-143-3p and subsequent inhibition of insulin receptor substrate 1 (IRS1) may lead to the initiation of NASH via the development of insulin resistance and hyperglycemia in the liver [130]. 

The expression dysregulated of several miRNAs also has been reported in many types of cancer, including NASH-associated HCC. Wang et al. demonstrated upregulation of oncogenic miR-155, miR-221/222, and miR-21, as well as downregulation of the most abundant liver-specific miR-122 at the early stages of hepatocarcinogenesis induced by the NASH model [134]. During the progression of NASH toward HCC, upregulation of miR-16 has also been observed. However, miR-16 and miR-15b were downregulated during the activation of HSCs, and the in vitro experiments confirmed that their upregulation significantly inhibited HSC proliferation, by increasing their apoptosis levels, and reduced hepatic fibrosis [135]. In addition, several miRNAs differentially expressed were reported in an HFD mouse model during the NAFLD-NASH-HCC transitions. Some miRNAs were overexpressed in tumors such as miR-155, miR-193b, miR-27a, miR-31, miR-99b, miR-484, miR-574-3p, miR125a-5p, miR-182, whereas others were downregulated in HCC, including miR-20a, miR-200c, miR-93, miR-340-5p, miR-720 [136]. MiR-155 has also been associated with the early stages of HCC [125,137]. As already mentioned, miR-122 is underexpressed in NASH patients [114], and was shown that deletion of miR-122 in mice resulted in hepatic steatosis, inflammation, fibrosis, and HCC [138]. MiR-21 is one of the most upregulated miRNAs in serum and hepatic tissues of patients with fibrosing-NASH and HCC [113]. In a NASH-related HCC model, the miR-21 expression was found significantly increased at the early stages of hepatocarcinogenesis [134]. Of note, dysregulation of miR-21 (upregulated) and miR-122 (downregulated) contributes to tumor invasion and metastasis in NASH-associated HCC [139]. Additionally, the dysregulation of miRNAs expression, usually attributed to the alteration of DNA methylation, could be involved as an accelerator of epigenetic instability [140]. Further research is necessary to validate the miRNAs dysregulated in NASH and NASH-HCC to use as noninvasive biomarkers. In summary, the main miRNAs dysregulated in NASH and NASH-HCC are described in Table 2, according to the references in the text.

#### 4.3.5. LncRNAs Involved in NASH and NASH-HCC

Long non-coding RNAs (lncRNAs, >200 nt) belong to non-coding RNA without or with limited protein-coding capacity. lncRNAs are generated through a pathway similar to that for protein-coding genes [141,142]. The lncRNAs are involved in several biological processes, including transcriptional regulation, mRNA splicing, and chromatin modification [143]. Atanasovska et al. identified 18 lncRNAs that responded to FFA/TNF-α and were associated with human NASH phenotypes, with most being related to inflammation. One novel intergenic lncRNA, designated lncTNF, was 20-fold upregulated upon TNF-α stimulation in HepG2 cells and positively correlated with lobular inflammation in the livers of NASH patients. Silencing lncTNF in HepG2 cells decreased NF-κB activity and suppressed expression of the NF-κB target genes such as *A20* and *NFKBIA* [144]. Metastasis-associated-lung-adenocarcinoma-transcript-1 (MALAT-1) is a lncRNA that promotes cell proliferation, migration, and invasion in several different human cancers, including HCC [145]. Hepatic levels of MALAT1 have been demonstrated to be overexpressed in NASH patients with fibrosis [146]. Another lncRNA, nuclear-enriched abundant transcript 1 (NEAT1), accelerates the progression of liver fibrosis and is associated with cell proliferation, invasion, and migration in HCC [147]. In addition, NEAT1 levels were found elevated in the liver tissue of NASH patients with advanced fibrosis [146]. In terms of hepatic fibrosis, a study of lncRNAs profiling in NASH patients identified specific pro-fibrotic lncRNA (lnc18q22.2, officially renamed as LIVAR) which was associated with NASH severity, lobular inflammation, and NAFLD activity score. Notably, the silencing of lnc18q22.2 expression resulted in reduced growth in HepG2 and IHH cells and caused cell death in Huh7 and Hep3B cells [148]. Maternally expressed gene 3 (MEG3) is a lncRNA located in the imprinted DLK1-MEG3 locus on human chromosome 14q32.3 region. MEG3 was reported significantly increased in human fibrotic and NASH cirrhotic liver [149]. lncRNAs have the potential as novel prognosis markers and targets for cancer therapy in the near future. 

#### 4.3.6. CircRNAs Expressed in NASH

Research circular RNAs (circRNAs) have garnered much interest recently due to their potential to serve as biomarkers or therapeutic targets. The majority of the current studies on circRNAs in liver diseases are focused on HCC and hepatitis, with limited information available for NASH [115]. Jin et al. evaluated a profile circRNAs expression in liver tissues of NASH mice, and they found that 69 circRNAs had increased expression, and 63 circRNAs had reduced expression. In addition, three of them have potential interaction with miR-122 as its sponge, thus affecting their respective genes in NASH (circRNA_002581-miR-122-Slc1a5, circRNA_002581- miR-122-Plp2, circRNA_002581-miR-122-Cpeb1) [150]. Identification of this circRNA/miRNA/mRNA interaction suggests a role of circRNAs in NASH. Therefore, the evaluation of circRNAs represents a promising strategy to evaluate and noninvasively monitor hepatic disease severity.

## 5. Pathogenesis of NASH-Related HCC 

### 5.1. Role of Lipotoxicity and Glucotoxicity in NASH and HCC Development 

Obesity is a strong independent risk factor for the development of cardiovascular disease and also confers an elevated risk of both hepatic and extra-hepatic complications [1,151]. NASH is considered a liver manifestation of metabolic syndrome associated with obesity, insulin resistance, T2D, and dyslipidemia [1,152]. Lipotoxicity and glucotoxicity are crucial for the development of simple steatosis in the liver and its progression to NASH, and these molecules favor fat deposition in the liver by different mechanisms such as mitochondrial defects, ER, and oxidative stress [90]. Insulin resistance and hyperinsulinemia stimulate the release of insulin-like growth factor 1 (IGF-1) and IRS-1, mediating signals for cell proliferation and inhibition of apoptosis and might contribute to the development of HCC [18]. In addition, the presence of obesity and insulin resistance has been associated with an increased risk for NASH and fibrosis [153]. High levels of AST and ALT are the most used markers for hepatic disease. The ratio of ALT/AST was reported to be a poor indicator of fibrosis with an accuracy of 0.66–0.74. However, the high levels of these enzymes do not correlate with the degree of fibrosis and are poor predictors of NASH when used in isolation [154]. Metalloproteinases and extracellular matrix by-products have been used as molecular biomarkers to confirm fibrosis, and to analyze fibrosis progression [154]. Although the molecular mechanisms are unclear, this is accompanied by drivers of disease, including liver oxidative stress, ER stress, inflammation, and among other drivers that further aggravate NASH with ballooning and fibrosis [1]. Hormones dysregulation plays an important role in NASH pathogenesis, such as adiponectin, leptin, and resistin. Adiponectin secreted from adipose tissue is negatively associated with insulin resistance, diabetes, and dyslipidemia because of its impact on fatty acid metabolism and insulin-receptor function [1,154]. Adiponectin is decreased in NASH, and its low levels have been found to be associated with fibrosis based on a meta-analysis [1,155]. Interestingly, adiponectin can inhibit tumor growth by either activating JNK-mediated mitochondrial apoptosis and caspases, or suppressing Akt and STAT3. However, low levels of adiponectin do not repress the Kupffer cells (KCs)-mediated inflammatory response and stimulate HCC development [156]. Leptin, resistin, and other pro-inflammatory mediators may cause adipose tissue dysregulation and a systemic insulin-resistant state [1]. Leptin is increased in obesity and is an important mediator of hepatic fibrosis in response to chronic liver injury, whether metabolic or toxic in etiology [1,157]. Leptin is also involved in the inflammatory response via triggering the JAK/STAT, PI3K/Akt, and ERK signaling pathways. Elevated serum leptin levels were reported in HCC patients with or without cirrhosis [156]. Additionally, leptin plays a carcinogenic role by increasing human telomerase reverse transcriptase (hTERT) expression in HCC liver tissues [158]. Furthermore, leptin-mediated neovascularization coordinates with vascular endothelial growth factor (VEGF) an important role in the development of liver fibrosis and hepatocarcinogénesis in NASH [159]. It is important to emphasize that HCCs are common hypervascular, including arterialization and sinusoidal capillarization. Angiogenesis is thought to result from imbalances in the VEGF, fibroblast growth factors, platelet-derived growth factors (PDGFs), angiopoietins, hepatocyte growth factor, endoglin (CD105) as well as the inhibitors of angiostatin, endostatin, thrombospondin-1, and among others [18]. Although the role of the observed dysregulated secreted factors remains unclear, osteopontin (OPN) and VEGF can promote HCC invasion [160]. Mice with OPN-deficient (Spp1^−/−^) showed enhanced hepatic lipid accumulation, increased hepatocellular apoptosis, and accelerated fibrosis, which aggravated NASH. However, the lack of OPN lowered systemic inflammation, prevented HCC progression to less differentiated tumors, and improved overall survival [161]. 

NASH mainly appears when the rate of liver non-esterified fatty acids (NEFA) uptake surpasses its capacity for esterification into triglycerides [162]. When NASH is already established, it can drive the development of fibrosis/cirrhosis and finally HCC, even simple hepatic steatosis may drive the development of HCC [48,163]. During obesity-related hepatocarcinogenesis, adipokines secretion is dysregulated, including the activation of the nuclear factor erythroid 2 related factor 1 (Nrf-1), nuclear factor kappa B (NF-κB), mammalian target of rapamycin (mTOR), phosphatidylinositol-3-kinase (PI3K)/phosphatase and tensin homolog (PTEN)/Akt, and Janus kinase/signal transducer and activator of transcription (JAK/STAT) signaling pathways [164]. The Hedgehog signaling pathway is also consistently activated in NASH and HCC [165]. In addition, fatty acid accumulation increases obesity-associated proteins in NASH-HCC, such as junctional protein associated with coronary artery disease (JCAD), and promotes activation of Yes-associated protein 1 (YAP1), which is essential for tumor growth [166]. In obesity and NAFLD/NASH was observed cardiolipin peroxidation, and a mitochondrial phospholipid modulates mitochondrial dynamics and morphology. The oxidated cardiolipin activates apoptotic processes by inducing cytochrome c release and represents a mitophagic signal to promote mitochondrial dismissal. Moreover, cardiolipin is frequently decreased during HCC progression as a possible strategy to avoid apoptosis [156]. Another player in the progression from NASH to HCC is fibroblast growth factor 21 (FGF21), which reduces fat deposition in the liver and acts as an inflammation suppressor. FGF21 also plays a key role in decreasing IL-17A levels and thus NASH development and transition to HCC [167]. 

### 5.2. Oxidative Stress in NASH-HCC

Oxidative stress and lipotoxicity play an important role in the progression of NASH and fibrosis [168]. The products of arachidonic acid oxidation and linoleic acid oxidation have been correlated among the oxidation products with NASH [154]. In NASH from an insulin-resistant obese model, reactive oxygen species (ROS) are generated during free fatty acid metabolism in microsomes, peroxisomes, and mitochondria. ROS are also generated by the presence of hepatic lipid peroxidation and mitochondrial dysfunction (structural mitochondrial lesions, decreased activity of the respiratory chain enzymes, and abnormal mitochondrial β-oxidation), which results in oxidative stress, hepatic hyperplasia, and in the development of HCC [1,169,170]. Moreover, the expression and activity of enzyme cytochrome P450 2E1 (CYP2E1), involved in hepatic microsomal fatty acid oxidizing and source for ROS production, has been reported elevated in both human and animal models of NASH [171]. NADPH oxidases (NOXs) are major producers of ROS, and NADPH oxidase 4 (NOX4) levels are increased in liver tissues of patients with NASH and mice with diet-induced steatohepatitis. NOX4 reduces the activity of the phosphatase PP1C, prolonging the activation of PKR and PERK-mediated stress signaling. Inhibitors of NOX4 decrease liver inflammation and fibrosis and ameliorate insulin sensitivity [104]. Interestingly, TAZ in pre-tumor NASH-hepatocytes, via induction of Cybb and NOX2-mediated DNA damage, contributes to the development of HCC tumors [104]. To note, the overexpression of hypoxia-inducible factor 1-alpha (HIF-1α) is promoted by intrahepatic ROS production. In NASH, oxygen is unevenly distributed in the hepatic lobules because of both inflammation and fibrotic scars, thus inducing hypoxia. Therefore, HIF-1α coupled with the release of inflammatory cytokines may mediate metabolic reprogramming, angiogenesis, and proliferation, thus prompting switching from NASH to HCC [156].

Oxidative stress is also elevated due to defects in redox defense mechanisms involving glutathione (GSH), catalase, or superoxide dismutase (SOD), which induces several events related to NASH and carcinogenesis, including DNA damage, tissue remodeling, and alterations in gene expression such as mutations in the *p53* tumor suppressor gene, which is observed in HCC [169,170]. For example, the mutagen 4-hydroxy-2-nonenal (4-HNE) has been associated with a mutation at codon 249 in the *TP53* gene [18]. Oxidative stress is implicated in blocking the expression of transcription factor Nrf1, which regulates gene transcription encoding enzymatic antioxidants. It was reported that disruption of Nrf1 function in the hepatocytes resulted in steatohepatitis and spontaneous development of liver cancer [170]. With respect to SIRT3 is found primarily in the mitochondria and is involved in oxidative stress to maintain mitochondrial integrity. Disruption of SIRT3 function in mice, either by genetic ablation or during high-fat feeding, has been reported to display metabolic syndrome and *Sirt3* knockout mice were documented to exhibit NASH. Both SIRT1 and SIRT3 are critical for redox state, epigenetic modification, and hepatic lipid metabolism to maintain the homeostatic equilibrium [172,173]. 

Excessive accumulation of fructose obtained by the Western diet induces uric acid, which generates ROS both in hepatocytes and adipocytes. Xanthine oxidase (XO) catalyzes the reaction converting xanthine into uric acid, and XO acts as an electron donor for oxygen, thus generating ROS and inducing a pro-inflammatory signaling cascade through the release of cytokines and oxidative stress in the liver [174]. ROS promotes inflammatory cytokines production such as TNF-α, IL-6, and leptin, thus preserving the inflammatory cascade and recruiting circulating monocytes and lymphocytes [11]. In high fat and cholesterol diet, the development of insulin resistance accelerates NASH and oxidative stress, aggravating liver inflammation. Cholesterol overload appears to contribute to the activation of KCs and HSCs. Excess cholesterol in ER lumen causes ER membranes disruption, thus inhibiting sarco/ER calcium ATPase (SERCA) activity, aggravating oxidative stress, mitochondrial dysfunction, ATP depletion, lipotoxicity, and hepatocyte degeneration, activating inflammatory cells and prompting the transition from simple steatosis towards NASH and fibrosis [175]. Permanent exposition of hepatocytes with oxidative and ER stress promoted by lipotoxicity and necroinflammation leads to cellular damage or cell death as well as disease progression resulting in immune cell infiltration, fibrogenesis, and activation of hepatic progenitor cells [151]. The unfolded protein response (UPR) and calcium extrusion from ER stores are commonly observed in NASH patients. Excessive calcium amount induces mitochondrial permeabilization, which enhances ROS production and caspases activation [175]. Oxidative stress from excess lipids increases caspase-2, causing hepatocyte apoptosis and probably resulting in compensatory hepatocyte proliferation [176]. Caspase-2 induces the activation of sterol regulatory element-binding proteins 1 and 2 (SREBP1/2), leading to triglycerides and hepatic-free cholesterol accumulation, respectively. Consistently, the expressions of caspase-2, SREBP1, and SREBP2 are elevated in liver samples from subjects with NASH [177]. With respect to hepatocyte transformation in addition to ER stress, other stressors are needed such as hypoxia, nutrient deprivation, and metabolic stress, which could alter UPR and prevent ER stress-induced apoptosis that might contribute to cancer cell aggressiveness [177]. Oxidative stress also promotes the activation of inflammatory pathways, including the expression of TNF-α, which leads to advanced fibrosis and cirrhosis [168]. Moreover, oxidative stress may also activate p62 under lipotoxicity conditions, which is involved in the p62-KEAP1-NRF2 pathway. Phosphorylated p62 triggers NFR2 expression in order to induce antioxidant defenses. Therefore, p62-KEAP1-NRF2 signaling may represent one of the pro-survival events for tumor initiation. Furthermore, the inhibition of ATG7 and beclin1 (autophagic proteins) may contribute to the escape strategy of ROS-induced apoptosis [156]. CD4^+^ T cells are selectively lost, these cells are important for antitumor surveillance and are susceptible to oxidative stress due to increased mitochondrial mass [176]. Therefore, oxidative stress affects immune signaling and contributes to carcinogenesis.

### 5.3. Chronic Inflammation in NASH-HCC

Hepatic insulin resistance and obesity are both well-established conditions, which induce systemic changes with chronic low-grade inflammation. Excessive fructose intake promotes the cascade of inflammatory pathways in hepatocytes and adipocytes, which may contribute to the activation of stress hormones involved in the hypothalamus-pituitary-adrenal, thus synthesizing immunosuppressors such as glucocorticoids [174]. NASH onset and progression are closely associated with liver inflammation that is partly regulated by the vagus nerve through α7 nicotinic acetylcholine receptor (α7nAchR). Kimura et al. reported that α7nAchR deficiency exacerbates hepatic inflammation and fibrosis in a mouse model of NASH [178]. In addition, the pro-inflammatory microenvironment in NASH creates a clinical condition that favors the onset of HCC [175]. Sterile inflammation occurs in the absence of pathogens, and is activated during NASH disease due to damage-associated molecular patterns (DAMPs) are released from damaged cells (e.g., nuclear and mitochondrial DNA, uric acid, and purine nucleotides), causing the maturation and secretion of both interleukin (IL)-1β and IL-8, thus sustaining inflammation [151,175]. Interestingly, sterile inflammation may induce the formation of neutrophil extracellular traps (NETs), structures composed of DNA-histone complexes and inflammatory proteins released by activated neutrophils, which has been shown to be important in chronic inflammatory conditions and in cancer progression. High elevated levels of NET markers have been reported in the serum of patients with NASH, suggesting that NETs are implicated in the pro-tumorigenic inflammatory environment in NASH [179]. Moreover, pathogen-associated molecular patterns (PAMPs), which include bacterial products such as LPS, are implicated in NAFLD or NASH liver injury [151]. DAMPs and PAMPs bind to pattern recognition receptors (PRRs) such as the Toll-like receptors (TLRs), which induce a local inflammatory response mediated by pro-inflammatory cytokines such as TNF-α and IL-6 [151]. TLRs are expressed in hepatic cells such as KCs, HSCs, biliary epithelial cells, and sinusoidal endothelial cells, as well as hepatic dendritic cells, being TLR2, TLR4, and TLR9 the most studied in NASH [151,180]. Indeed, during hepatic inflammation are increased the number of hepatic macrophages and the expression of TNF-α, IL-6, and C-C motif chemokine ligand 2 (CCL2) [181]. Consistent with these findings, obesity-promoted HCC development is dependent on enhanced production of TNF-α and IL-6 (tumor-promoting cytokines), which aggravate hepatic inflammation [11]. TNF-α and IL-6 in turn may activate pro-oncogenic pathways via c-Jun N-terminal kinase (JNK) and activator of STAT3, Janus kinase 2 (JAK2), mitogen-activated protein kinase (MAPK), and PI3K [11,182]. TNF-α and IL-6 also promote the compensatory growth of hepatocytes through NF-κB, mTOR, and STAT3 in response to mitochondrial-induced apoptosis and preserve a pro-survival tumor microenvironment via the paracrine/autocrine release of chemokines (CCL2, CCL7, and CXCL13) and cytokines (TNF-α, IL-1β, IL-6) [156]. Interestingly, an androgen receptor (AR)-driven oncogene named cell cycle-related kinase (CCRK) contributes to obesity-induced pro-inflammatory signaling, inducing the development of NASH-related hepatocarcinogenesis. CCRK induces transcription activation of STAT3-AR promoter that subsequently activates mTORC1/4E-BP1/S6K/SREBP1 cascades via GSK3β phosphorylation. Furthermore, STAT3-AR-CCRK-mTORC1 pathway components have been reported to increase NASH-associated HCC [103]. In this context, inflammation and STAT3 contribute to HCC initiation, progression, metastasis, and immune suppression. In addition, STAT3 phosphorylated levels in hepatocytes and HSCs correlated with the severity of NASH (lobular inflammation, ballooning inflammation, and advanced fibrosis) [18]. In mice, models were reported that loss or ablation of TNF-α and IL-6 prevents fat-induced hepatic injury and development of HCC [11]. To note, HSCs can be activated by cytokines and chemokines, such as TNF-α, IL-1β, IL-6, TGF-β1, and CCL2 and 5, secreted from hepatocytes and KCs [156]. HSCs activated produce collagen and fibrotic scars, stimulating apoptotic receptors, such as Fas, TNF receptor 1 (TNFR1), and TNF-related apoptosis-inducing ligand (TRAIL) on hepatocytes surface [156]. Consequently, the pro-apoptotic B-cell lymphoma 2 (Bcl-2) and JNK pathways promote mitochondrial permeabilization through their translocation on mitochondrial outer membranes, creating tunnels in which apoptosis-inducing factor (AIF) and cytochrome c are released, inducing mitochondrial derangement and apoptosis [183]. With respect to this, RIP1- and RIP3-activated JNK have been proposed as an apoptotic pathway implicated in the appearance of liver injury, inflammation, and fibrosis in both NASH patients and the NASH mouse model [184]. In addition, KCs produce other pro-inflammatory cytokines such as IL-12 and IL-18; and these cells act to stimulate fibrogenic responses via the production of TGF-β1, MMPs, PDGF, and ROS. In the mouse model, the depletion of KCs using clodronate significantly decreases the severity of NASH [180]. Other pro-inflammatory mediators from the liver with NASH are expressed, including high-sensitivity C-reactive protein (hs-CRP), fibrinogen and plasminogen activator inhibitor 1 (PAI-1), and several acute-phase proteins [185]. In addition, chronic liver inflammation in NASH stimulates tissue remodeling and fibrogenesis, causing perisinusoidal fibrosis, which can progress to bridging fibrosis and cirrhosis. Particularly, hepatic fibrosis is the main predictor of morbidity and mortality in subjects with NASH [186]. The 4-HNE compound is an end-product of lipid peroxidation and has potent pro-fibrogenic effects by inducing the expression of genes involved in extracellular matrix deposition, which is the main mechanism of fibrosis and cirrhosis [156]. Moreover, cirrhosis is a well-established risk factor for HCC, but it is important to note that NASH-related HCC can develop in the absence of cirrhosis or fibrosis [187]. Inflammatory markers, including IL-6, IL-8, TNF-α, TGF-β1, CRP, and ferritin have been involved in the recruitment of neutrophils and initiation of hepatocellular inflammation, and also associated with hepatic fibrosis liver as well as damage and progression to NASH [77,154]. 

NLRP3 inflammasome is an intracellular multiprotein complex implicated in the production of mature IL-1β, promotion of metabolic inflammation, and progression of NASH in both mice and patients [188]. NLRP3 is also responsible for the maturation of IL-18 and activates immune system modulators implicate in stimulating fibrosis and inflammation [174]. NLP3 inflammasome promotes the activation of inflammatory pathways and the re-establishment of hepatic homeostasis, by activating apoptosis. NLP3 is completely lost or downregulated in HCC patients, thus favoring the compensatory proliferation of hepatocytes and HCC onset [189]. In summary, continual stimulation of fibrotic and inflammation damages hepatocytes, leading to cirrhosis and HCC. 

### 5.4. Mitochondrial Dysfunction Plays a Key Role in the Transition from NASH to HCC

Mitochondria are vital for cell homeostasis because they provide energy requirements through oxidative phosphorylation (OXPHOS) and ATP synthesis, also regulate redox status, β-oxidation, tricarboxylic acid cycle (TCA), ketogenesis, and glucidic/lipidic metabolism [156]. The excessive accumulation of fatty acids increases β-oxidation and ROS production, which alter mitochondrial energy metabolism pathways. The mitochondrial abnormalities are accompanied by reduced intracellular antioxidant protection in NASH, altering fatty acid metabolism that can cause metabolic stress [190]. The transport of excess FFAs into the mitochondria increases in addition to β-oxidation, TCA cycle flux, and oxidative phosphorylation, contributing to ROS generation, lipid peroxidation, apoptosis, and inflammation [191]. Importantly, the mitochondrial β-oxidation of fatty acids can be either increased (insulin resistance-associated NASH) or decreased (drug-induced NASH) [192]. Both fructose and the Western diet may hasten mitochondrial depolarization and mitophagy, which lead to mitochondrial dysfunction at the early stages of NASH, thus contributing to NASH progression towards HCC [193]. The increased intracellular Ca2^+^ concentration increases ROS levels and promotes the mutagenesis of both nuclear DNA and mitochondrial DNA (mtDNA), thus promoting the activation of oncogenes or the inhibition of onco-suppressors, and altering mtDNA replication [156]. The mutations caused by NASH affect the development of insulin resistance, the rate of hepatic fat buildup, and promote metabolic reprogramming, including blunted ketogenesis and increased glycolysis in the presence of oxygen, which may increase the risk of NASH-associated HCC [174].

Mitochondrial biogenesis (mitobiogenesis) is the process by which cells renew from pre-existing ones, involving fusion and fission events, since they cannot be generated de novo [156]. In the liver tissues of several NASH subjects, a disequilibrium of mitobiogenesis was observed and the accumulation of damaged mitochondria due to the failure of mitophagy, and NASH was reported to correlate with alterations in mitochondrial architecture [194]. PGC1α protects against NASH progression by promoting an anti-inflammatory environment by balancing the numbers of M1 and M2 macrophages [195]. However, PGC1α upregulation may contribute to HCC development by coordinately sustaining mitochondrial biogenesis and β-oxidation [196]. Type 1 mitophagy is completely related to nutrient availability and insulin signaling, whereas type 2 mitophagy may either occur in parallel with PINK1/Parkin-dependent mitophagy or be promoted by photodamage in a phosphoinositide 3-kinase (PI3K)-independent manner [156]. Lipidomic analysis of NASH patients has revealed that high levels of dihydroceramide and dihexosylceramide species are correlated to mitochondrial dysfunction, oxidative stress, and inflammation [197,198]. Furthermore, the accumulation of dihexosylceramide was found in human HCC tissue, suggesting that decreased mitophagy may be implicated in the progression from NASH to HCC [199]. Fusion-fission unbalancing alters mitochondrial architecture and leads to the formation of megamitochondria. In a cohort of 31 biopsied NASH patients, megamitochondria with crystalline inclusions were distributed randomly among zones and without variation in abundance, regardless of the fibrosis stage [200]. In another study made in pediatric NASH, was found loss of mitochondrial cristae, and the presence of linear crystalline inclusions within the mitochondrial matrix of an increased electron density, which were distributed randomly both within the hepatic parenchymal cell and the zones of the hepatic lobule, suggested a major role of these organelles in the morphogenesis of pediatric NASH [201]. Therefore, mitochondrial dysfunction plays a key role in the NASH-to-HCC transition.

### 5.5. Dysregulation of Autophagy in NASH-Derived HCC 

Autophagy is a lysosomal degradative pathway necessary for cell survival due to providing energy in stressful conditions. Autophagy plays a critical role in the progression of NASH and HCC. In hepatic tissue, autophagy suppresses protein aggregate, lipid accumulation, oxidative stress, chronic cell death, and inflammation. As a selective autophagy, lipophagy is a mechanism of lipid metabolism and, in hepatocytes, suggests that impaired lipophagy may contribute to the development of NASH [202]. CD36 promotes hepatic steatosis and is a negative mediator of autophagy, thus contributing to NASH pathogenesis [203]. Autophagy plays a double-edged sword role in NASH and NASH-associates HCC. In the early phase of tumor development, autophagy suppresses tumorigenesis and removes any damaged or mutated cells. However, once a tumor is well-established, autophagy cells promote tumor growth and contribute to HCC survival [154].

The induction of autophagy through the increased expression of liver-specific Atg7 in ob/ob mice improved metabolic stress and reduced hepatic steatosis. The loss of beclin-1, an autophagy gene, leads to HCC development. The autophagy inhibited in NASH leads to the accumulation of p62/SQSTM1 (p62), an autophagic substrate hypothesized to be implicated in the formation of the Mallory-Denk bodies (MDB). MDB are present in ballooned hepatocytes and are markers for NASH diagnosis [204]. The impairment of autophagy in tumor cells due to metabolic stress, results in the accumulation of p62, leading to elevated retainment of damaged mitochondria, enhancing oxidative stress and DNA damage [205]. In addition, p62 is involved in the activation of the NF-κB-signaling pathway, including the transcription of genes encoding for antioxidant proteins and detoxification enzymes through the activation of the nuclear factor erythroid 2-related factor 2 (Nrf2) [204]. TGF-β-activated kinase 1 (TAK1) is a positive regulator of AMPK, and its deletion in a mouse model led to increased mTOR activity and the suppression of autophagy with severe hepatic steatosis. Furthermore, these animals developed spontaneous hepatocarcinogenesis, with high levels of p62 in the hepatocytes. However, autophagy activity was restored by rapamycin with both the downregulation of mTOR and the attenuation of hepatocarcinogenesis development [206]. Collectively, these findings suggest that NASH-HCC development is related to defects in the autophagy pathway.

### 5.6. Immune Dysregulation in NASH-HCC

The innate and adaptive immune mechanisms are important components of steatosis, insulin resistance, inflammation, and fibrosis during NASH progression. Innate immune cells, such as macrophages are essential players in the progression of NASH and have been intensively investigated [207]. In the early stages of inflammation associated with NASH, both macrophages and lymphocytes are the most common inflammatory infiltrates in lever tissue [173]. In NASH the hepatic macrophage polarization changes to the M1 state [208]. Tim-3 functions as an immune checkpoint in the regulation of both adaptive and innate immune cells, including macrophages. Tim-3 serves as an important predictor in MCD-induced NASH by negatively regulated the production of ROS and related downstream pro-inflammatory cytokine secretion of IL-1β and IL-18 in macrophages [209]. In addition, macrophages are critical in HCC pathogenesis. M1 macrophages produce pro-inflammatory and immunostimulatory cytokines (IL-1β, TNF-α, INF-β1, among others) to participate in antigen presentation, thus with anti-tumorigenic activity [210]. Moreover, M2 macrophages which are activated by IL-4 or IL-13, produce anti-inflammatory and immunosuppressive effectors (Arg-1, IL-10, CD206, among others), which exert pro-tumorigenic effects [211]. Tumor cells from HCC can polarize infiltrating macrophages to M2-tumor-associated macrophages (TAM), while at the same time suppressing M1-TAM polarization, which leads to the declination of cytotoxic T cells (CD8^+^ T cells) in tumor tissues [212]. Therefore, M1 phenotype is a potential strategy for HCC treatment. Platelet cargo, platelet adhesion and platelet activation are important for NASH and onset hepatocarcinogenesis. In especially, platelet GPIbα is an intermediary in the hepatic immune cell trafficking and antiplatelet therapy (e.g., aspirin/clopidogrel and ticagrelor), prevented NASH and HCC development [213].

Adaptive immunity involves T and B cells which play a role in the triggering and progression of NASH. Mechanistically, livers with NASH may accumulate B cells with increased pro-inflammatory cytokine secretion and antigen presentation ability following activation through the innate adaptor myeloid differentiation primary response protein 88 [186]. B cell activating factor (BAFF) is required for survival and maturation of B cells. High BAFF levels have been demonstrated in serum of NASH patients, and the serum BAFF level correlates with B cell expression in the liver [214]. TNF-α induces intercellular adhesion molecule-1 (ICAM-1) expression in liver and injures hepatocytes by causing the adhesion of activated CD8^+^ T cells to hepatocytes [215]. In NASH patients have been reported high levels of soluble ICAM-1 [216]. CD8^+^ T cells have been also reported accumulated in the liver of patients with NASH, correlating with elevated frequency of blood CD8^+^ T cells expressing perforin, IFN-γ, and TNF-α, thus leading to NASH-related HCC. Experimental depletion of CD8^+^ T cells in animal models improves NASH and NASH-associated HCC, restoring hepatic insulin sensitivity, decreased liver damage, and reduced fibrosis [186]. CD4^+^ T cells have a potential role in promoting NASH through the release of pro-inflammatory cytokines such as IFN-γ [217]. Supporting a potential role for CD4^+^ T cells in NASH, whole-body IFN-γ deficiency attenuates infiltration of intrahepatic macrophages as well as disease progression in a mouse model [218]. In addition, the potential role of CD4^+^ T cells in hepatocarcinogenesis was demonstrated in several animal models [184]. Th17 cells infiltration (produce IL-17) is found in NASH livers, and IL-17 exacerbates hepatic steatosis and inflammation. Furthermore, IL-17 signalling upregulates the expression of pro-fibrotic genes in HSC, while its absence reduces the levels of pro-inflamamtory cytokines and cell death in a murine model of liver fibrosis [173]. 

TNF receptor superfamily member 4 (TNFRSF4, also known as OX40) deficiency inhibits Th1 and Th17 differentiation, as well as suppresses monocyte migration, antigen presentation and M1 polarization. TNFRSF4 levels in serum are positively associated with NASH, and it is a key regulator of both intrahepatic innate and adaptive immunity and is involved in the development of NASH [188]. Additionally, the progression to NASH is characterized by an increment of the Th17/regulatory T cells (Tregs) ratio in peripheral blood and liver [173]. However, it was showed that Tregs increase in the liver during NASH, and their depletion inhibits the transition from NASH to HCC in a choline-deficient, high-fat diet plus diethylnitrosamine mouse model [219]. Therefore, possibility the Tregs have opposite functions in early and late NASH with the subsequent hepatocarcinogenesis.

**Figure 2:**
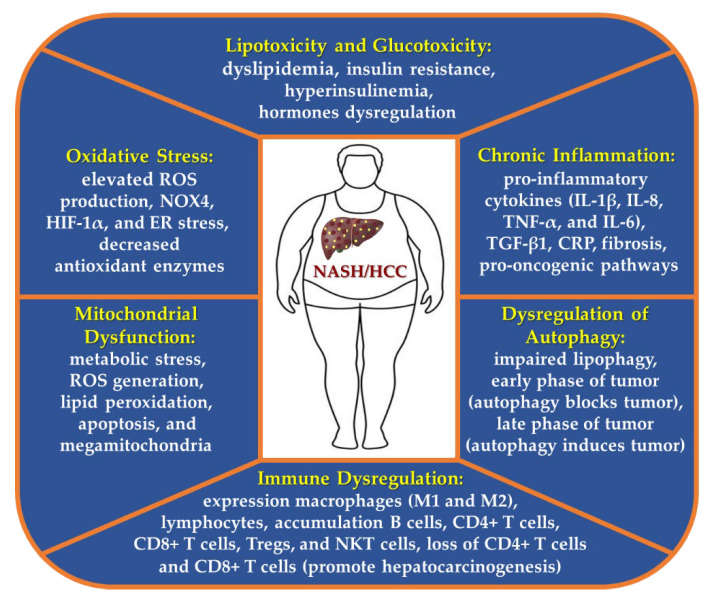
Pathogenic processes implicated in the onset and development of NASH-related HCC. Hyperlipidemia and hyperglycemia caused by obesity, promote insulin resistance and hormones dysregulation, which are responsible to trigger oxidative stress, inflammation, mitochondrial dysfunction, as well as dysregulation of autophagy and immune function, leading to NASH and ultimately HCC. Abbreviations: CRP, C-reactive protein; ER, endoplasmic reticulum; HIF-1α, hypoxia-inducible factor 1 alpha; NKT, natural killer T; NOX4, nicotinamide adenine dinucleotide phosphate reduced (NADPH) oxidase 4; ROS, reactive oxygen species; Tregs, regulatory T cells.

Several studies indicate that natural killer T (NKT) cells display detrimental effects during NASH development, and this cells can be activated in response to elevated levels of IL-12, type I interferons and IL-1 during NASH [173]. NKT cells have been shown to promote fibrosis during NASH using CD1d-deficient mice fed an MCD diet [220]. In mice deficient in NK cells administrated with a high fructose diet, developed less steatosis, suggesting that NK cells may be implicated in NASH development [221]. To note, NK cells also inhibit the development of fibrosis through direct killing of early and senescent activated HSC [173]. In addition, T-cell receptor (TCR)-invariant lymphocytes recognizing lipid species, have been found to favor lipid storage in hepatocytes by the secretion of LIGHT (a member of TNF family also known as TNFSF14). NKT cells and CD8^+^ T cells accentuate liver damage through the production of IFN-γ, generating a permissive microenvironment favoring the emergence of transformed NASH hepatocytes. By contrast, CD4^+^ T cells exhibit protective effects in limiting both tumor initiation and tumor progression [177]. Therefore, there are several biological processes dysregulated that promote the transition from NASH to HCC, thus determining the severity of HCC disease (Figure 2). 

## 6. Metabolic Reprogramming in NASH and NASH-Related HCC 

### 6.1. Alteration of Carbohydrate Metabolism in HSC

The liver plays a central metabolic role and metabolic disturbances are early events implicated in the development of chronic hepatic diseases and HCC [222]. In NASH patients, elevated ROS levels are induced due to persistent increase in activity of TCA cycle, despite reduced β-oxidation, insufficient lipid esterification, and impaired ketogenesis [223,224]. Liver fibrosis may be present in NASH, and is characterized by an excessive and imbalanced deposition of fibrous extracellular matrix (ECM), which is mainly formed by collagens (type I and type III), fibronectin, elastin fibers, among other. The main secretors of ECM proteins are activated myofibroblasts. In normal conditions, HSCs are found in a quiescent state where they function as pericytes and reservoirs of retinol (vitamin A). When liver is injury, HSCs are transdifferentiated to myofibroblasts, which secrete TGF-β1, producing collagens and establishing an extracellular matrix [225,226]. HSCs in transdifferentiation state (activated HSCs) have enhanced glycolytic flux compared to quiescent cells [227]. 

Carbohydrate metabolism is a complex biochemical process, which ensures a constant supply of energy to living cells. Glycolysis metabolizes glucose to pyruvate, following to its transformation to either lactic acid (anaerobic glycolysis) or to acetyl-CoA (aerobic glycolysis), which is utilized in the tricarboxylic acid cycle (TCA) and subsequently occurs oxidative phosphorylation. Activated HSCs were reported to shunt their glycolysis pathway towards the production and accumulation of lactate, despite increased expression of the lactate transporter MCT4 and increased lactate efflux [225,227]. Interestingly, the inhibition of lactate accumulation converted myofibroblasts to quiescent HSCs [228]. Therefore, activated HSCs acquire an evolutionary advantage that allows them to prioritize ATP production rate over efficiency (i.e., ATP yield per glucose molecule) [227]. However, activated HSCs still require the most energy from oxidative phosphorylation because of their increased number and activity of mitochondria reported [225]. Additionally, primary culture-activated or immortalized rat HSCs show higher levels of glucose transporter proteins, including GLUT1, GLUT2, and GLUT4, as well as proteins implicated to the intracellular processing of glucose, such as hexokinase 2 (HK2), fructose-2,6-bisphosphatase-3 (PFKFB3), and pyruvate kinase (PK), thus HSCs obtain more energy to be fully active [225]. In addition, activated HSCs express downregulated proteins of gluconeogenesis, such as phosphoenolpyruvate carboxykinase-1 (PCK1) and fructose bisphosphatase-1 (FBP1) [228]. Pyruvate dehydrogenase kinase 3 (PDK3) is upregulated in activated HSCs and favor to the lactate accumulation from pyruvate [228]. However, these findings come from in vitro experiments, thus they need to be confirmed in vivo models. 

### 6.2. Carbohydrate Metabolism in HCC

Metabolic reprogramming is widely recognized as one of the hallmarks of cancer [9,195]. The transformation of hepatocyte cells (normal liver) to tumor cell occurs along with changes of the gene expression and cellular metabolism [229]. Hexokinase 2 (HK2) is the first enzyme implicated in glycolysis. *HK2* is expressed in skeletal and cardiac muscle and adipose tissues [230]. The enzymes included in glycolysis (e.g., HK2 and pyruvate kinase M2, PKM2) are highly expressed in HCC and tumor cells, leading to Warburg effect [6,231], due to the energy demand for rapid cell multiplication, tumor cells catabolize glucose into lactate through glycolysis, despite the availability of oxygen for aerobic metabolism (mitochondrial oxidative phosphorylation), tumor cells need the energy for the synthesis of nucleic acids, proteins, and lipids as components of the new transformed cells [232]. With respect to this, cancer cells promote aerobic glycolysis in the neighboring stromal cell, which produce lactate, fatty acids, amino acids, glutamine, and ketone bodies that are used by maintain growth of cancer cells [174]. The tumor cells acquire several advantages when obtain its energy utilizing glycolysis; for instance, they do not produce mitochondrial ROS, reduce capacity to undergo apoptosis, and increase glucose uptake, thus allowing tumor cells to grow rapidly and out-compete healthy tissue for available glucose [233]. In response to metabolic stress, cancer cells may change from glycolysis and primarily utilize the pentose phosphate pathway (PPP), which produces NADPH, a coenzyme for lipid synthesis and ribose-5-phosphate generation (a precursor for nucleotide synthesis) for multiplying tumor cells. NADPH is implicated in the generation of glutathione (GSH), a potent antioxidant that detoxifies ROS and carcinogens, which are accumulated in active and metabolically stressed cancer cells [174]. Additionally, increased GSH levels are associated with elevated HCC proliferation and growth and contributes to chemo-resistance [174]. The enzymes representative for PPP are glucose-6-phosphate dehydrogenase (G6PD) or transketolase (TKT), respectively. Both enzymes G6PD and TKT are upregulated and associated with clinicopathological features of HCC. Furthermore, G6PD contributes to migration and invasion of HCC cells in vitro by promoting epithelial-to-mesenchymal transition (EMT) through the STAT3 pathway [6]. Mechanistically, epidermal growth factor receptor (EGFR) induce the PI3K/Akt signaling pathway and consequently promote GLUT1 expression and the activity of phosphofructokinase-2 (PFK2), which stimulates glycolysis by activating PFK1 and in turn inhibits gluconeogenesis through suppressing fructose 1,6-biphosphatase 1 (FBP1) [174].

#### Gluconeogenesis and Tricarboxylic Acid Cycle Are Reduced in HCC

Gluconeogenesis, a reverse process of glycolysis, synthetize glucose from pyruvate, lactate, glycerol, and gluconeogenic amino acids [234]. In HCC tissues and cells the gluconeogenesis enzymes are decreased, including FBP1 and phosphoenolpyruvate carboxykinase (PCK1) that are also associated with poor prognosis in patients with HCC [6]. Hepatic glucose-6-phosphatase alpha deficiency promotes to autophagy impairment, mitochondrial dysfunction, enhanced glycolysis, and elevated PPP, which contributes to hepatocarcinogenesis [6]. When cells are subjected to glucose starvation, both p53 and PGC1α are activated in an AMPK-dependent pathway. Furthermore, the AMPK-p38-PGC1α axis provides a growth advantage to cancer cells by inducing oxidative metabolism via augmented mitochondrial biogenesis and oxidative phosphorylation [195]. PGC1α have been implicated in NASH-HCC pathogenesis, due to can regulate several metabolic pathways, such as gluconeogenesis, mitochondrial biogenesis, as well as FA oxidation, the antioxidant response, and de novo lipogenesis (DNL) [195]. 

Lactate is the main carbon source for the TCA cycle in cancer cells [235]. Pyruvate dehydrogenase A1, the main active subunit of pyruvate dehydrogenase, is decreased in HCC tumor tissues and is positively correlated with survival rate of HCC patients. Pyruvate dehydrogenase A1 overexpression inhibits glycolysis and promotes oxidative phosphorylation, increasing apoptosis through a mitochondria-dependent pathway [236]. In line, isocitrate dehydrogenase 2 (IDH2) is reduced in HCC tissues and has a strong negative correlation with metallopeptidase 9 (MMP9). IDH2 deficiency promotes cell migration and HCC metastasis [237]. Malic enzymes (MEs) catalyzes the oxidation of TCA cycle intermediate malate to form pyruvate and CO2, reducing NAD(P)+ to NAD(P)H. MEs is critical for glycolysis, gluconeogenesis, fatty acid synthesis and glutamine metabolism. ME1 is a poor prognostic predictor of HCC because of its silencing decreases HCC metastasis via inhibition of EMT through ROS-induced pathways [6]. Metabolic pathway involved in carbohydrates metabolism could be a new therapeutic target for chemoprevention of NASH-HCC, although further studies should be investigated.

### 6.3. Role of Lipid Metabolism in NASH-HCC

Liver is main organ in lipid metabolism and generates apolipoproteins, endogenous lipids, and lipoproteins [238]. Accumulation of triglyceride and diacylglycerol in the liver is a hallmark of NASH [1]. Children with NASH have high levels of apolipoprotein B to apolipoprotein AI (ApoB/ApoAI). Atherogenic dyslipidaemia is strongly associated with NASH, thereby may predict elevated cardiometabolic disease risk [239]. In early stages of steatosis, mTORC1 is increased to support lipid synthesis and export. Whereas in NASH, mTORC1 activity is decreased, which may promote to reduced phosphatidylcholine synthesis and VLDL-triglycerides export, thus promoting inflammation and fibrosis [240]. Hepatic and systemic insulin resistance increase of FFAs flux within the liver, as consequent of dysregulation of the lipolysis-lipogenesis equilibrium, to the decreased lipoprotein export and to inhibition of lipophagy, thereby resulting lipotoxicity give rise to a chronic damage of hepatic tissue [241]. Excessive FFAs lead to increase β-oxidation and ROS production, limiting mitochondrial function [242]. Additional to mitochondrial abnormalities, the diminution intracellular antioxidant protection in NASH along with alteration of pathways of fatty acid metabolism can cause metabolic stress [190]. In response to oxidative stress, hepatocytes make changes to FFAs oxidation. For instance, carnitine palmitoyltransferase 2 (CPT2) expression is decreased, which helps hepatocytes to resist lipotoxic effects but also promotes hepatocarcinogenesis [243]. In addition, UCP2 activity is increased due to FA surplus, thereby increasing mitochondrial proton leak and in turn NASH susceptibility [244]. Other metabolic causes of hepatic steatosis, include long-chain FAs (LCFAs) β-oxidation within peroxisomes and ω-oxidation in ER, both overexpressed in NASH; and defects in the mitochondrial trifunctional protein (MTP), which catalyzes β-oxidation of LCFAs, promoted hepatic insulin resistance and steatosis development, including an increase in antioxidant defenses and cytochrome P-450 to counteract ROS production [156,241]. In summary, hepatic steatosis is associated with defects in intrahepatic lipolysis (e.g., decreased ATGL/CGI-58 activity), defects in triglyceride export (e.g., defective Apo-B 100, MTTP activity), augmented glucokinase activity resulting in elevated hepatic DNL, and reductions in hepatic mitochondrial/peroxisomal β-oxidation [245].

With respect to lipolysis in HCC, three major enzymes are involved in this process to release of fatty acids, such as adipose triacylglycerol lipase (ATGL), hormone-sensitive lipase and monoacyglycerol lipase (MAGL) [246]. MAGL promotes progression of HCC via NF-κB-mediated EMT process [247]. Moreover, the overexpression of CD36 promotes EMT transition in HCC [248]. ATGL is highly expressed in HCC tissues and hydrolyzes the triacylglycerols into diacylglycerol and FFAs, generating high levels of these metabolites, which indicate poor prognosis [246]. FFAs are energy-generating nutrients, signaling molecules, structural components of the cell membrane, which are essential for cancer cell proliferation. Normal cells preferentially use circulating exogenous lipids, while HCC cells have a high rate of de novo lipid synthesis. Furthermore, cancer cells showed increased uptake of FAs and FAs β-oxidation [7]. Fatty acid-binding protein 1 (FABP1), specific of liver, is responsible for the transportation of long chain FAs and plays important roles in intracellular lipid metabolism. Both FABP1 and vascular endothelial growth factor receptor (VEGFR) expression are overexpressed in HCC. FABP1 promotes HCC cell migration through VEGFR2/SRC proto-oncogene tyrosine-protein kinase signaling and focal adhesion kinase/cell division cycle 42 pathway, inducing angiogenesis, tumorigenesis and metastasis. FABP5 overexpression is associated with invasion and metastasis in HCC tumor tissues [6]. 

The enzymes of lipogenesis are increased in various cancers, including HCC, such as ATP citrate lyase (ACLY), acetyl-CoA carboxylase (ACC), fatty acid synthase (FASN), acyl-CoA synthease (ACS) and stearoyl-CoA-desaturase 1 (SCD1) [6]. ACC is the first and rate-limiting enzyme in DNL, which transforms Acetyl- CoA to Malonyl-CoA, and its activity inhibited by AMPK [249]. Liver-specific ACC inhibitor ND-654 impedes liver DNL, inflammation and development of HCC [250]. Cancer cells utilize cytoplasmic acetyl-CoA as a substrate for FA synthesis. Therefore, malonyl-CoA and acetyl-CoA are condensed by FASN to form palmitate with the presence of NADPH and other fatty acid synthesis products. In addition, knockdown or pharmacological inhibition of *FASN* blocked the growth of HCC in vitro [6,7]. SCD catalyzes the conversion of saturated fatty acids into monounsaturated fatty acids (MUFAs), mainly palmitoleate (C16:1) and oleate (C18:1) from palmitate (C16:0) and stearate (C18:0), respectively. Thus, the accumulation of MUFAs and SCD signaling are involved in HCC progression [6,7]. The effect of SCD1 in HCC is related with the regulation of P53, WNT/b-catenin, EGFR and autophagy [6]. SCD1 plays a critical role in regulating liver tumor-initiating cells and sorafenib resistance via modulation of ER stress-mediated differentiation. Agents targeting SCD1 in combination with sorafenib is a promising treatment strategy against liver cancer [251]. The transcription factor SREBP1 is significantly high in HCC tumor and is central player in cellular FA metabolism and controls the expression of various lipogenic enzymes, including ACC, FASN, and SCD [7]. SREBP1c is the common isoform in HCC and in the normal liver. Knockout of *SREBP1* in HCC cells induced growth arrest and apoptosis, as well as inhibited the migration and invasion of tumor cells, whereas the overexpression of SREBP1 promoted cellular proliferation, suggesting that SREBP1 may be a therapeutic target for HCC [6,7]. Liver X receptor (LXR) is a member of ligand activated nuclear receptor superfamily of transcription factors. LXRα and LXRβ regulate cholesterol homeostasis and lipogenesis by transactivation of SREBP1 and FASN. In addition, LXRα is downregulated in HCC tumor tissues, and LXR expression correlates with liver fat deposition, liver inflammation and fibrosis [252]. Liver X receptor (LXR) is an oxysterol-activated nuclear receptor involved in the control of cholesterol homeostasis and lipogenesis. In human liver, farnesoid X receptor (FXR) negatively regulates lipid synthesis and mice lacking FXR are more susceptible to develop NASH probably due to the induction of the inflammatory and fibrogenic response mediated by NF-κB signaling pathway. Moreover, FXR null mice develop HCC, which may be associated to elevated levels of IL-1β and activation of Wnt/βcatenin and c-myc [252,253]. Lipid metabolism reprogramming in mesenchymal cells is orchestrated by β-catenin and these cells mainly use exogenous FAs for triacylglycerol synthesis by diacylglycerol acyltransferase 1 (DGAT1) [254]. Therefore, acumulation of saturated triacylglycerols and phospholipids, as well as reduction of ceramides and polyunsaturated fats in HCC tissues provides protection to the tumor cells from apoptosis and oxidative stress [255]. In well-differentiated HCC, the expression levels of enzymes related to glycolysis, PPP, FA synthesis are increased, while tricarboxylic acid (TCA) cycle and β-oxidation are suppressed [9]. Consistently, levels of ketone bodies are reduced in patients with NASH compared to individuals with simple steatosis [256]. Therefore, metabolic reprograming (e.g., increased glycolysis and reduced β-oxidation) plays a role key in the increase the risk of NASH-related HCC. 

### 6.4. Cholesterol Plays an Important Role in the Transition from NASH to HCC

**Figure 3:**
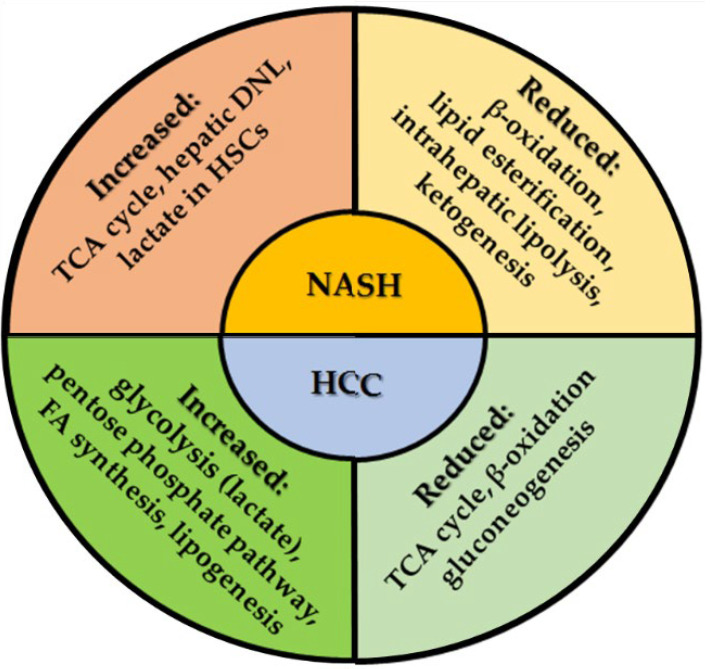
Dysregulation of metabolic pathways in NASH and HCC. There is an increase in the TCA cycle and lactate (HSCs) production in NASH, while β-oxidation, lipid esterification, and intrahepatic lipolysis are decreased, leading hepatic steatosis. The tumor cells express an increase of glycolysis with production of lactate (Warburg effect), elevated pentose phosphate pathway (nucleotide synthesis for multiplying tumor cells), FA synthesis and lipogenesis. The tumor cells also show reduced TCA cycle, β-oxidation and gluconeogenesis, indicating that metabolic reprograming plays a key role in hepatocarcinogenesis. Abbreviations: DNL, de *novo* lipogenesis; FA, fatty acid; HSCs, hepatic stellate cells; TCA, tricarboxylic acid.

Cholesterol is important for cell structure and cell proliferation. The cholesterol content in the Western diet is 10-fold higher than in the Mediterranean diet [58]. Several studies suggest that cholesterol is a risk factor for NASH and high levels of free cholesterol promotes inflammatory and fibrotic pathways in the liver [257]. Free cholesterol is directly fibrogenic toward stellate cells [258]. Cholesterol tended to suppress CPT activity and adenosine triphosphate-binding cassette transporter G5 (ABCG5) expression. A high-fat and high-cholesterol diet induces hepatic features of NASH and eventually progresses to cirrhosis in Sprague-Dawley rats within 9 weeks [259]. Cholesterol is involved in several biochemical pathways which are potentially important in HCC development, such as several cytokine and signaling pathways [260]. For instance, IL-6, TNF-α, and IL-1 inhibit triglyceride synthesis [261]. IL-6 and TNF-α cytokines not only induce insulin resistance in metabolic tissues, but also promote carcinogenesis [182]. The consumption of cholesterol is 2-fold higher in HCC tissues compared with normal tissues [262]. Cholesterol is important for membrane integrity and fluidity, as well as is required for highly proliferative cancer cells, including HCC [6]. In summary, consumption of lipid enriched diets, especially cholesterol, could elevate the risk of HCC development with a severe phenotype. The altered metabolic pathways are outlined in Figure 3. 

## 7. Epigenetic Modifications in NASH and HCC

### 7.1. DNA Methylation Patterns in NASH

Methylation is a heritable enzyme-mediated chemical transformation and is catalyzed by DNA methyltransferases (DNMTs), which transfers a methyl group from S-adenosyl methionine (SAM) to carbon 5 of the Cytosine ring. DNA methylation mainly occurs in the Cytosine base (C) when is followed by a Guanine (G), thereby named CpG sites. In general, when methylation occurs in the gene promoter region results transcriptional repression, while methylation in the gen region promotes gene expression, as described in most cancers including HCC [5,263]. The only enzymes that participate in DNA methylation are DNMT1, DNMT3a and DNMT3b. DNMT1 is essential for maintaining DNA methylation in the genome during DNA replication, while DNMT3a and DNMT3b are responsible for de novo methylation of DNA, methylating DNA from unmethylated DNA, and are crucial for the cell development and differentiation [263]. 

Nutrition is closely related to DNA methylation since this epigenetic modification depends on the availability of SAM, which requires methyl donors from food (folate, choline, methionine, vitamin B12, and betaine) for its synthesis [173,264]. In this respect, methyl-deficient diets accompanied by change in the expression of hepatic DNA methyltransferases cause liver injury, including NASH [173]. Previous studies have reported that long-term administration of a MCD lacking methyl donors induced global DNA hypermethylation, resulting fat liver and fibrosis [119,265]. Marked global DNA hypomethylation, aberrant DNA and histone (H3K9 and H3K27) methylation at several genes were reported in steatosis and NASH developed from mice fed with methyl-deficient diet [266]. Therefore, DNA methylation is considered a key factor in the progression from simple steatosis to NASH. In patients with NASH exhibit higher hepatic expression of DNMT1 and a concurrent augment in DNA methylation of the mitochondrial NADH dehydrogenase 6 (MT-ND6), causing to ultrastructural defects in mitochondrial morphology [267]. Importantly, aberrant DNA methylation in NASH can lead to silencing of genes implicated in DNA repair, lipid metabolism, glucose metabolism and progression of fibrosis. In especially, the epigenetic changes in the gene encoding chromodomain helicase DNA-binding protein 1 (CHD1), which are reported associated in the transition from NASH to HCC [268]. Moreover, augmented methylation at a CpG site (cg11669516) and reduced gene expression of insulin-like growth factor binding protein 2 (*IGFPB2*) are common reported in mice and patients with NAFLD and NASH [269]. Additionally, hypomethylation of a CpG site within or proximal to the activating transcription factor (ATF)-binding motif of hepatic genes is related with increased expression of these genes involved in glycolysis (*PFKL*), DNL (*ACACA, FASN*), and insulin signaling (*PRKCE*) in obese and T2D patients [270]. As already mentioned, PGC1α protects against NASH progression and is encoding by *PPARGC1A* gene, and its promoter methylation is increased in adult patients with NASH compared to those isolated steatosis, correlating with insulin resistance, reduced mitochondrial DNA, and decreased gene transcriptional activity [263]. SIRT1-deacetylated PGC1α activates PPARα target genes implicated in hepatic fatty acid oxidation and ketogenesis [269]. In addition, liver fibrosis is associated with changes in DNA methylation density at the *PPARG* promoter analyzed in circulating cell-free DNA obtained from patient plasma [271]. PPARγ is an antifibrotic and adipogenic nuclear receptor, its expression must be silenced for HSC to activate and acquire their myofibroblast phenotype and is realized by two epigenetic mechanisms. First, methylated CpGs within the promoter of *PPARG* recruit MeCP2, which promotes repressive H3K9me3-modifying enzymes to inhibit initiation of transcription. Second, transcriptional elongation is blocked by enhancer of zeste homolog 2 (EZH2, is the catalytic subunit of polycomb repressive complex 2)-mediated H3K27me3 modifications in the downstream coding region of *PPARG* gene, with expression of EZH2 being dependent on MeCP2. EZH2 is a histone H3K27 methylation leads to recruitment of the PRC1 Polycomb complex which causes chromatin condensation, and promotes hepatic fibrosis by repressing the transcription of *PPARG*. Conversely, MeCP2 has also been shown to stimulate the transcription of several pro-fibrotic genes through the control of ASH1, an H3K4/H3K36 histone methyltransferase that directly binds to the regulatory regions of *αSMA, collagen 1, TIMP1* and *TGF-β1* in activated HSCs, thus promoting their transcription [272,273,274]. Alternatively, overnutrition leads to the activation of steatotic target genes of PPARγ2 through association with the histone methyltransferase MLL4, providing an important link between diet and epigenetic factors [275]. It was also reported that a differentially methylated region in the *PNPLA3* promoter was hypermethylated in the livers of more severe (F3-4) fibrotic NASH, and was inversely correlated with mRNA levels, significantly with the GG genotype [271]. Smaller study from a Chinese population found 6 CpG sites located in the *CSL4, CRLS1, CTP1A, SIGIRR, SSBP1*, and *ZNF622* genes, which are differentially methylated in peripheral blood leukocytes of patients with NASH compared with those exhibiting simple steatosis. However, only differences in DNA methylation of *ACSL4* were confirmed by pyrosequencing [276].

### 7.2. Aberrant DNA Methylation in HCC

Alterations in DNA methylation and its machinery are commonly linked with cancer, including HCC. In cancer occurs three types of aberrations in DNA methylation: hypermethylation of the CpGIs in the promoter regions of tumour suppressor genes, altered expression of DNMTs, and global hypomethylation of genes and repetitive sequences, thereby leading to genomic instability and oncogene activation [5]. A study reported that EGFR, estrogen receptor 1 (ESR1), and glycine N-methyltransferase (*Gnmt*) genes were inhibited and concurrently methylated in HCC, and also hypermethylated in NASH with advanced fibrosis. This result supports the hypothesis of simultaneous accumulation of CpG island methylator phenotype alterations in the disease process causing to HCC [277]. The majority of studies in HCC have been reported significantly hypermethylated genes, such as *CDKN2A, RASSF1, APC* and *SMAD6*, while other studies described hypomethylated genes, such as *IGF2, CCL20*, and *NQO1*; as reviewed by [5]. 

Methylation of lysines on histones is regulated by several methyltransferases and demethylases, which is an important mechanism of epigenetic histone modifications. Generally, histone 3 lysine 4 methylation (H3K4me) is related with gene activation, while histone 3 lysine 9 methylation (H3K9me) and histone 3 lysine 27 methylation (H3K27me) lead in gene repression [5]. With respect to this, 11 methyltransferases and demethylases, such as EZH2, EHMT2, SETDB1 and SETD2 were reported associated with the clinical characteristics of tissues from HCC patients [278]. H3K9 methylation is commonly associated with the pathogenesis of HCC, in which the global levels of H3K9me2 and H3K9me3 are frequently higher in cancer tissues, and both H3K9me2/3 correlated with the degree of tumor differentiation and with poor prognosis [279]. In human HCC the overexpression of EZH2 correlated with augmented levels of focal adhesion kinase (FAK) and H3K27me3. This finding evidenced a new molecular nexus between EZH2 and FAK, which may control HCC growth, and also has been correlated with poor prognosis in pediatric HCC [5]. Histone lysine N-methyltransferase SUV39H2, an enzyme capable of adding mono, di, and trimethylated labels to H3K9, plays an important role in the activation of inflammatory pathways. In addition, it can decrease the activity of SIRT1 inducing NASH progress. *SUV39H2* activity was investigated in knockdown mice fed a HFD, and they exhibited a less severe form of NASH compared with the control mice for this enzyme [280]. It was published that the development of NASH-related HCC showed a global loss of histone H4 lysine 20 trimethylation (H4K20me3), as well as global and gene-specific deacetylation of histone H4 lysine 16 (H4K16). Histone H4K16 deacetylation resulted in the silencing of genes associated to the cell death that occurred during the development of NASH-related HCC [281]. Therefore, changes in DNA methylation and histone modification are necessary to sustain tumor growth. 

### 7.3. Histone Modifications Involved in HCC

Histone proteins undergo various modifications such as acetylation, phosphorylation, methylation, ubiquitination, SUMOylation, and ribosylation, among which acetylations have been largely reported. Histone acetylation generated by the histone acetyltransferase (HAT) activates the gene transcription, whereas histone deacetylation catalyzed by histone deacetylase (HDAC) promotes the gene repression [172]. p300 (a HAT) mediates acetylation (H3K9ac) after phosphorylation induced by EGF in HCC cells. In this signalling pathway there are an overexpression of high-mobility group protein A2 (HMGA2), commonly upregulated in HCC and associated with metastasis and poor survival, and deficiency of p300 reversed the EGF-induced HMGA2 expression and histone H3K9ac [282]. HAT1 is overexpressed in HCC specimens respect to normal liver tissues. HAT1 promotes glycolysis in HCC cells, and its protein level is augmented with tumor stage [283]. Another HAT, called males absent on the first (MOF) is important in the transcription activation by acetylating histone H4K16. MOF was demonstrated decreased in HCC, correlating with poor overall survival. *MOF* knockdown promotes HCC growth, while its overexpression reduces HCC growth, partially through promoting of the SIRT6 expression [284]. H4K16ac have been identified as a biomarker of microvascular invasion in HCC, and MOF downregulation caused a decrease of expression in genes involved in vascular invasion in HCC cells, reducing tumor cell intravasation and metastasis [285]. Histone phosphorylation has been associated with active gene transcription. Aurora kinases phosphorylate most of serine/threonine residues of the histone H3. Aurora A (centrosome-associated) is commonly increased in HCC patients, correlating with high grade tumors. Aurora-A decreases radiotherapy-induced apoptosis by activating NF-κB signaling, thus contributing to HCC radioresistance [286].

HDAC8 is activated by SREBP1, an insulin-responsive transcription factor, which activates the transcription of lipogenic genes, and HDAC8 deficiency in obesity-associated mouse models of NASH and HCC ameliorated insulin resistance, reducing triglyceride levels and tumour growth. Mechanistically, HDAC8 interacted with the chromatin modifier EZH2 to concordantly inhibit Wnt antagonists via histone H4 deacetylation and H3 lysine 27 trimethylation [287]. Furthermore, downregulation of EZH2 reduced HCC cell growth, partially through the inhibition of β-catenin signaling. Conversely, ectopic overexpression of EZH2 in immortalized hepatocytes activated Wnt/β-catenin signaling to promote cellular proliferation [288]. Additionally, silencing of HDAC4, HDAC6, and HDAC8 expression repressed TGF-β1-mediated α-SMA transcript levels; the most profound repression was observed with HDAC4 silencing, which resulted in an almost complete failure of TGF-β1-treated fibroblasts to overexpress α-SMA transcript and protein [289]. Interestingly, miR-137 is decreased in HCC cell lines and have an inhibitory effect on HCC migration and invasion in vitro. EZH2 is a direct downstream target gene of miR-137 in HCC and miR-137 suppresses invasion and migration by targeting EZH2-STAT3 signaling in HCC cells in vitro and in vivo [290]. On the other hand, FASN acetylation is frequently decreased in human HCC, and correlates with high levels of HDAC3. In addition, an HDAC3 inhibitor destabilizes FASN proteins and inhibits the growth of HCC [291]. 

Another epigenetic marker implicated in HCC progression is the Ubiquitin-specific protease 22 (USP22), in charge of the deubiquitination of both histones H2A and H2B. Histone deubiquitination is commonly associated to transcriptional activation, epigenetic regulation and cancer development [5]. In HCC have been reported high levels of USP22 expression, which correlate with clinical stage, tumor grade and shortened survival time [292]. Consistently, different studies reported that USP22 levels were associated with drug-resistant phenotype of HCC [5]. Furthermore, the H2B deubiquitination by ATXN7L3 protein increased SMAD7 expression, which acts to suppress tumor growth in HCC [293]. 

SUMOylation consist in the reversible addition of a small ubiquitin-like modifier (SUMO) group to proteins. This modification have profound effects on carcinogenesis and multidrug resistance in HCC. In the human genome the SUMO isoforms (SUMO-1, SUMO-2, and SUMO-3) are encoded. SUMO-1 has been found highly expressed in HCC cell lines and HCC specimens, and SUMO-2 expression is robustly correlated with patient survival rate [5]. Moreover, SUMO-1 can alter the activity of methyltransferase-like 3, inducing HCC progression via regulating Snail (an epithelial to mesenchymal transition effector) mRNA homeostasis [294]. SUMO-2 also regulates liver kinase B1 (LKB1) and exportin-5 (XPO-5) oncogenic activity in HCC [295,296].

**Figure 4:**
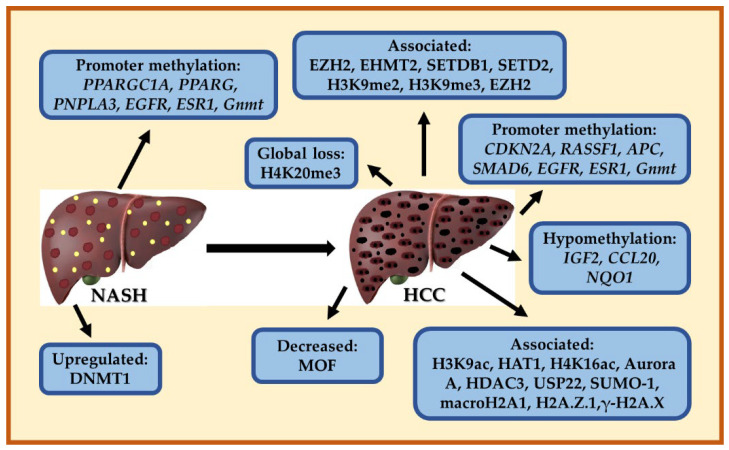
Representative epigenetic modifications in NASH and HCC. DNMT1 is essential for maintaining DNA methylation and is upregulated in NASH. Several genes have their promoter methylated in NASH and HCC. Other histone and mark modifications are involved in HCC, which are necessary to sustain tumor growth. Abbreviations: APC, adenomatosis polyposis coli; CCL20, C-C motif chemokine ligand 20; CDKN2A, cyclin dependent kinase inhibitor 2A; DNMT1, DNA methyltransferase 1; EGFR, epidermal growth factor receptor; ESR1, estrogen receptor 1; IGF2, insulin-like growth factor 2; Gnmt; glycine N-methyltransferase; NQO1, NAD(P)H: quinone oxidoreductase 1; MOF, males absent on the first; PNPLA3, patatin-like phospholipase domain-containing protein 3; PPARG, peroxisome proliferator activated receptor gamma; PPARGC1A, peroxisome proliferator-activated receptor gamma coactivator-1-alpha; RASSF1, ras association domain family member 1; SMAD6, SMAD family member 6; USP22, ubiquitin-specific protease 22.

Histone variants are non-canonical (non-allelic) variants of histones, showing one or a few amino acid differences and affect chromatin remodeling and histone post-translational modifications. For instance, in vitro studies demonstrated a potential oncogenic role for H2A.Z isoforms in hepatocarcinogenesis [5]. In HCC patients and in HCC cell lines H2A.Z.1 was significantly overexpressed, correlating with poor prognosis. H2A.Z.1 also induced proliferation by selectively modulating tumor microenvironment regulatory proteins such as E-cadherin and fibronectina, which were elevated. *H2A.Z.1* knockdown suppressed HCC cell growth, and reduced the metastatic potential of HCC cells by selectively regulating cell cycle components [296]. Histone variant macroH2A1 contains a domain that shows 66% homology with histone H2A, this variant may contribute to different cellular processes, such as cell cycle regulation, stem cell differentiation, and DNA repair and transcription in somatic and cancer cells. There are two alternatively exone-spliced isoforms, macroH2A1.1 and macroH2A1.2 [5]. The protein levels of macroH2A1 splice variants were reported upregulated in the livers with HCC of animal models and patients [297]. It has been shown that *macroH2A1* knockdown cell lines acquire cancer stem cells features, such as enhancement of the tumorigenic potential, slow proliferation, resistance to chemotherapy treatments, elevating in mRNA expression of reprogramming genes, and augmented glycolysis, reflecting the same phenotype of human undifferentiated and aggressive HCCs, which express a low level of macroH2A1 [298]. Furthermore, macroH2A1 was able to reprograms carbohydrate and lipid metabolism of HCC cells towards cancer stem cells with elevated lipid accumulation through triggering of the LXR pathway [299]. Phosphorylation of the Ser139 residue of the histone variant H2A.X, generating γ-H2A.X, is an early cellular response to the induction of DNA double-strand breaks. Several authors have been reported an increase of phosphorylated histone variant γ-H2A.X in HCC tissue, which can be used as potential indicator for HCC surveillance [5]. Taken together, transition of NASH-HCC implicates various cellular processes governed by accumulation of epigenetic changes, favoring activation of oncogenes that inactivate tumor suppressor genes and disrupt cellular homeostasis (Figure 4).

## 8. Current Pharmacological Therapies and Emerging Therapies for NASH/HCC

### 8.1. Therapeutic Strategies for Preventing and Treating of NASH

There are currently no therapies indicated for use in patients with NASH, despite its prevalence and clinical significance. Although several drugs are in development with potential therapeutic and effective at reversing the histopathologic features of NASH, difficulties in identification of accurate biomarkers have retard the approval of effective therapeutics. A continuation are described summary current therapeutic strategies and pharmacological therapies for NASH.

#### 8.1.1. Lifestyle Modification and Exercise

Lifestyle modification have been recommended as the main method for the nonclinical management of NASH, with several publications reporting on this. Since 1970, studies indicated that weight loss improves liver histology as evidenced by biopsies and liver enzyme levels [13]. In general, a weight loss of 3–5% improves insulin sensitivity and steatosis. Whereas a greater weight loss of 7–10% ameliorates all features of NASH, including steatosis, lobular inflammation, ballooning, and fibrosis. However, when patients regained weight, fibrosis worsened [13,300]. In addition, consumption of a diet with calories less (Mediterranean diet) can reduce body weight, hepatic lipid accumulation, and insulin resistance, including decreased serum levels of saturated fatty acid and increased serum levels of monounsaturated and n-3 polyunsaturated fatty acid [301]. 

Long-term exercise can prevent NASH development by promoting the phagocytic capacity of liver resident KCs and decreasing liver inflammation and fibrogénesis, including reducing risk of diabetes, hypertension and metabolic syndrome [302,303,304]. In fact, vigorous activity is more beneficial for NASH and fibrosis than moderate activity [304]. In mouse model was reported that maternal exercise can reduce Western diet-derived obesity and ameliorate hepatic lipid metabolism via activating the AMPK and PGC1α signaling pathways [305]. To note, diet and exercise have beneficial effects in cardiovascular risk factors and causing to body weight reduction [306]. Therefore, lifestyle modification based on dietary changes, such as caloric restriction (dietary composition) and exercise are important recommendations for the nonclinical management of patients with NASH.

#### 8.1.2. Vitamin E (Antioxidant) 

Oxidative stress is implicated in the progression of NASH and the hypothesis that an antioxidant agent as vitamin E (α-tocopherol) can attenuate liver histology has been tested in NASH experimental protocols [307]. Several effects of vitamin E treatment have been reported in patients with NASH, such as reduced serum transaminase activities and TGF-β1, as well as hepatobiliary enzymes, hepatic steatosis, inflammation, and hepatocellular ballooning compared with the control group. However, fibrosis improvement was not confirmed in this studies and comparison of reported results was limited because of different inclusion criteria and doses of vitamin E, as well as use of other drugs, and limited histological data [300,307]. A study from Japan with long-term vitamin E treatments (300 mg/day) for more than 2 years reported that histological steatosis, inflammation, and fibrosis did not change after treatment, but liver fibrosis improved in seven patients (approximately 41%) [308]. The findings suggest that metabolic, genetic and epigenetic factors could influence the effectiveness of vitamin E in the NASH patients. Currently, EASL and AASLD guidelines recommend vitamin E (800 IU daily) as a potential short-term treatment option for nondiabetic adults with biopsy-proven NASH despite a potential increase in all-cause mortality, hemorrhagic stroke, and prostate cancer that has been reported [13,307].

#### 8.1.3. Pharmacological Treatment Aimed at Reducing Steatosis and Glucose Levels in NASH

##### Thiazolidinediones

Thiazolidinediones (TZDs) including pioglitazone, troglitazone, and rosiglitazone are a family of anti-diabetic medications, and they are high-affinity ligands for PPARγ [309]. Pioglitazone showed an improvement in steatosis and inflammation, but with smaller improvement in fibrosis in patients with NASH [300]. However, variable efficacy was reported, a study of 247 non-diabetic adults with NASH to receive pioglitazone, vitamin E or placebo, for 96 weeks. Pioglitazone did not meet its primary endpoint, serum alanine and aspartate aminotransferase levels were decreased (*p* < 0.001), and there was also a reduction in hepatic steatosis (*p* < 0.001) and lobular inflammation (*p* = 0.004), but not with improvement in fibrosis scores (*p* = 0.12) [310]; suggesting the use of pioglitazone may be limited in NASH. However, in a meta-analysis was reported that pioglitazone improved advanced fibrosis in NASH, even in patients without diabetes [311]. Conflict results have been reported about long-term safety of pioglitazone, a narrative review reported that was associated with an elevated risk of heart failure, bone fracture, oedema, and weight gain. However, another narrative review published that pioglitazone reduced the risk of major cardiovascular events, such as myocardial infarction, stroke, and cardiovascular death, suggesting that the long-term safety profile of pioglitazone remains to be established in NASH patients [300].

##### Liraglutide

Liraglutide is a glucagon-like peptide-1 (GLP-1) receptor agonist. Liraglutide is effective at improving indices of glycemic control, and has been approved for use in the United States, Europe, and Japan for the treatment of T2D [312]. Also, it improves hepatic steatosis in mice with diet-associated NASH [313]. Liraglutide was investigated in 52 patients in a phase II double-blind RCT in patients with NASH from four United Kingdom medical centers, with the primary outcome being resolution of NASH and no deterioration of fibrosis [314].

##### Metformin

One of the first antidiabetic drugs evaluated in NASH was metformin, which is a first-line anti-diabetic drug of the biguanide family. Metformin reduces hepatic glucose production and promotes peripheral glucose utilization. It also attenuates insulin resistance via activation of the AMPK pathway and inhibits the mitochondrial glycerophosphate dehydrogenase shuttle with change in redox state [315]. A comparison between metformin, vitamin E, and diet alone showed aminotransferases levels normalization, decreasing liver fat, necroinflammation, and fibrosis in a limited number of patients [316]. However, it is not recommended for the treatment of NASH without diabetes, due to other trials have not confirmed these results in children and adolescents with NAFLD and NASH [13]. Although metformin does not ameliorate NASH, there is evidence that it might decrease the incidence of HCC [317].

##### Obeticholic Acid

Obeticholic acid (OCA), a synthetic variant of natural bile acid chenodeoxycholic acid, is a ligand of FXR. In animal models, FXR activation reduces hepatic glucogenesis, lipogenesis, and steatosis [307]. Clinical results of OCA, showing improvements in steatosis, inflammation, and fibrosis for patients with noncirrhotic NASH, including patients with NASH and comorbid T2D [300,307]. However, these improvements were related with significant increases of LDL-cholesterol (bad cholesterol) in patients with noncirrhotic NASH [300]. A study showed that OCA-induced increases in LDL-cholesterol in patients with NASH were mitigated with atorvastatin [318]. In terms of adverse events, who received 25 mg of OCA experienced pruritus in half of patients, with severe intensity in 28% [319]. Therefore, it will be necessary to confirm efficacy and safety of OCA in the next studies. 

##### Statins

Statins have anti-oxidant and anti-inflammatory effects due to their lipid lowering action [320]. A prospective study included 31 patients with biopsy-proven NASH showed that atorvastatin (10 mg daily) for 2 years improved lipid levels, liver function, adipocytokines levels, fibrosis markers, long-chain fatty acid composition, and liver histologic findings. However, 4 patients experienced progression of fibrosis over the 2-year period [321]. With respect to simvastatin appeared to have no effect on serum aminotransferases, hepatic steatosis, necroinflammatory activity or stage of fibrosis in patients with NASH [322]. However, in patients with NASH and dyslipidemia treated for 12 months with pitavastatin 2 mg/day, therapy showed an improvement of the metabolic parameters and even histology in some patients [323]. A cross-sectional study showed that patients either NAFLD or NASH treated with statin had poorer glycaemic control despite more frequent antidiabetic therapy than those without statins, and fibrosis was not different between statin users and non-users [324]. Interestingly, in a multi-center cohort of 1201 European individuals was reported that the protective effect of statins on steatohepatitis was stronger in subjects not carrying the I148M PNPLA3 risk variant (*p* = 0.02 for interaction), thus the I148M PNPLA3 risk variant limited this beneficial effect [325]. Based on meta-analysis, statin use is associated with a reduced risk of HCC, most strongly in Asian but also in Western populations [326]. Additionally, statin use reduce the risk of HCC among patients with diabetes [327]. A retrospective study evaluated the chemoprotective effect of statin use in patients with NASH-related advanced liver fibrosis (bridging fibrosis [F3] and cirrhosis [F4]). Statin use was associated with a lower risk of developing HCC in patients with NASH cirrhosis [328].

##### Omega-3 Polyunsaturated Fatty Acids (PUFAs)

Omega-3 polyunsaturated fatty acids (PUFAs) include α-linolenic acid (ALA; 18:3 ω-3), stearidonic acid (SDA; 18:4 ω-3), eicosapentaenoic acid (EPA; 20:5 ω-3), docosapentaenoic acid (DPA; 22:5 ω-3), and docosahexaenoic acid (DHA; 22:6 ω-3). Several experimental and epidemiological studies have shown that the ω-3 PUFAs decrease the risk of cancer, which is also supported by several clinical studies [329]. Capanni et al. reported improvements in transaminases, triglycerides, and fasting glucose with long-term supplementation of n-3 PUFAs [330]. A subsequent trial confirmed the beneficial effects of PUFAs in the improving ALT levels and liver echo-texture on ultrasound [331]. Another study reported that treatment with 4 g/day for 15 to 18 months of DHA plus EPA decreased liver fat, but no improvement in the fibrosis scores occurred, thereby PUFAs failed to show therapeutic benefit in patients with NASH/NAFLD [332]. Since 2002, prescription agents containing EPA+DHA or EPA alone have been approved by the FDA to treat hypertriglyceridemia [333]. 

#### 8.1.4. Pentoxifylline (Anti-Inflammatory)

Pentoxifylline (PTX), a methylxanthine derivative, is an inhibitor of TNF-α and decreases oxidative stress by increasing hepatic glutathione synthesis [307]. Zein et al. showed that PTX therapy over 1 year significantly improved steatosis and lobular inflammation in patients with NASH [334]. Another study on 30 patients treated with PTX 1200 mg/day for 1 year, demonstrated similar findings with a lowering in transaminases and improvement in steatosis and hepatocellular ballooning in patients with NASH when compared to placebo [335]. However, a meta-analysis of five studies showed that PTX improved histological findings such as lobular inflammation and NAS without affecting lipid profiles [336]. Further investigations with large randomized trials are required to determine its effect on lipid profiles.

#### 8.1.5. Antifibrotic Drugs

Hepatic fibrosis grade is the most important determinant of mortality in NASH patients [307]. Therefore, there is a need to find effective antifibrotic agents mainly for patients with advanced fibrosis. Several antifibrotic drugs have been developed for the treatment of fibrosis in NASH. 

##### Cenicriviroc

Cenicriviroc (CVC), a C-C motif chemokine receptor-2/5 (CCR2/5) antagonist, has anti-inflammatory and antifibrotic effects, and also improves insulin sensitivity [307]. CCR5 antagonist is predicted to reduce the migration, activation, and proliferation of collagen-producing HSCs [337]. Antifibrotic effects of CVC were a confirmed in a diet-induced mouse model of NASH, in which CVC reduced fibrosis despite ongoing steatohepatitis [338]. A randomized, double-blind, multinational phase 2b study reported in NASH patients that after 1 year of CVC treatment, this drug achieved improvement in fibrosis. Although asymptomatic amylase elevation (grade 3) was observed more commonly in the CVC group than placebo group, this drug is well tolerated [339]. The authors reported the final data from year 2 exploratory analyses, corroborating antifibrotic findings from year 1. Indeed, the majority of patients treated with CVC who achieved fibrosis response at year 1 maintained it at year 2, with greater effect in advanced fibrosis [340]. Phase 3 evaluation for the treatment of subjects with NASH and stage F2 or F3 fibrosis is now ongoing and recruiting (AURORA study; NCT03028740).

##### Pirfenidone

Pirfenidone (PFD) is an orally bioavailable pyridone derivative, which has antifibrotic, anti-inflammatory, and anti-oxidative effects, and clinically used for the treatment of idiopathic pulmonary fibrosis [341,342]. PFD inhibited TNF-α and prevented liver injury and fibrosis associated with decreased apoptosis of liver cells in Western diet-fed melanocortin 4 receptor-deficient (MC4R-KO) mice [343]. In a mouse NASH model induced by high-cholesterol and high-fat diet, PFD reverted hepatic insulin resistance and steatohepatitis by polarizing M2 macrophages, partially through attenuation hepatic lipid accumulation and peroxidation, as well as by reducing the expression of genes related to lipogenesis and fatty acid synthesis and promoting the expression of those related to fatty acid oxidation [344]. In addition, we reported that PFD is an agonistic ligand for PPARα and improves clinical characteristics of NASH by activation of SIRT1/LKB1/pAMPK [345]. We also published that PFD have cardioprotective effects by preventing obesity-induced cardiac steatosis and fibrosis in mice with NASH [346]. PFD is efficacious and safe, reducing advanced liver fibrosis in 35% of patients analyzed [347], thereby PFD can be repositioned as an antifibrotic agent for human NASH.

### 8.2. Current Pharmacological Therapies and Treatment Options for NASH-HCC

The development of HCC involves dysregulation of several biological processes such as cell cycle, apoptosis, and many other cellular pathways. Tumor cell progression is promoted by mutations in various proteins implicated in the regulation of cell cycle. Therefore, the research for the treatment of HCC is focused on proteins target such as CDKs or growth factors to suppress the tumor development [348]. The progression to cirrhosis can occur in approximately 25% of NASH patients, which elevates the risk of HCC [349]. When HCC has been established in the cirrhosis liver, the prognosis and the therapeutic approach depend on both tumor stage and liver function (Child-Pugh score) [15]. The FDA has approved first and second-line treatment for HCC and all these new therapeutic strategies have substantially revolutionized the treatment for advanced HCC, which are discussed a continuation.

#### 8.2.1. First-Line Treatment for HCC 

##### Bevacizumab Plus Atezolizumab

In 2020, the combination of bevacizumab and atezolizumab was approved by the FDA, thereby is the new standard of care for the systemic first-line treatment in unre-sectable HCC [15]. Atezolizumab is a monoclonal antibody, which blocks programmed cell death ligand-1 (PD-L1) and bevacizumab is a monoclonal antibody against VEGF [350]. In a phase Ib trial, the treatment of atezolizumab (1200 mg) with bevacizumab (15 mg/kg) intravenously every 3 weeks was safe in patients with advanced HCC, which showed a median follow-up of 6.6 months and median progression-free survival (PFS) was 5.6 months [351]. Based on these promising results, an open-label phase III trial (IMbrave150), patients with unresectable HCC with previously untreated, were randomly assigned in a 2:1 ratio to receive either atezolizumab plus bevacizumab or sorafenib, which resulted an overall survival at 12 months was 67.2% with atezolizumab-bevacizumab and 54.6% with sorafenib, and a median PFS was 6.8 months compared to 4.3 months in the respective groups [352]. In addition, updated overall survival analysis for IMbrave150 (after 12 months of additional follow-up), which enrolled 501 systemic treatment-naive patients with unresectable HCC, 336 to atezolizumab plus bevacizumab and 165 to sorafenib. The median overall survival was also improved to 19.2 months with combination therapy and 13.4 months for sorafenib, and with the same median PFS [353]. Another study reported a similar median of PFS (6.5 months) in patients with advanced HCC from Germany and Austria. However, in patients with compromised liver function (Child B and C), the treatment with atezolizumab and bevacizumab showed low efficacy [354]. Furthermore, atezolizumab and bevacizumab therapy is associated with comparable efficacy and tolerability in older age patients (age ≥ 65 years) with unresectable HCC [355]. Interestingly, a retrospective analysis of prospectively collected data from consecutive patients with non-viral advanced HCC (Italy, Japan, Republic of Korea, and UK), treated with atezolizumab plus bevacizumab (190 patients), lenvatinib (569 patients), or sorafenib (210 patients), showed that lenvatinib was associated with a longer overall survival in the whole population, even lenvatinib treatment was associated with a longer overall survival and PFS in the NAFLD/NASH population compared to atezolizumab plus bevacizumab. Whereas in the subgroup of non-NAFLD/NASH patients, no difference in overall survival or PFS was observed [356]. Therefore, these findings suggest that lenvatinib is associated with a significant survival benefit in patients with NAFLD/NASH-derived HCC. 

##### Sorafenib

Sorafenib is a multikinase inhibitor targeting PDGF-A/B, VEGFR1-3, c-kit, RET, and various proteins of the kinase cascade, Ras, C-Raf, B-Raf, ERK, FLT-3, JAK/STAT as well as MAPK, contributing to its antitumor effect by decreasing tumor cell proliferation, inducing apoptosis, and suppressing angiogenesis [14,348]. In 2007, sorafenib was approved based on the results of Sorafenib HCC Assessment Randomized Protocol (SHARP) trial and Asia-Pacific trial [357,358]. Both trials demonstrated that there was a small but significant median overall survival benefit with sorafenib in advanced HCC (SHARP: sorafenib: 10.7 months versus the placebo: 7.9 months; Asia-Pacific: sorafenib: 6.5 months versus the placebo: 4.2 months) [357,358]. Sorafenib showed generally manageable adverse events, the most common grade 3/4 adverse events included hand-foot skin reaction (SHARP: 8%; Asia-Pacific: 11%) and diarrhea (SHARP: 8%; Asia-Pacific: 6%) [357,358]. Despite sorafenib increases survival in advanced HCC patients, drug resistance is commonly encountered through several mechanisms, including hypoxia-inducible factors (HIFs) [359]. A study evaluated 504 HCC patients treated with sorafenib (Study-1), and overall survival of HCC patients who received sequential molecular-targeted agents’ (MTAs) therapy after first-line sorafenib was significantly longer (the median survival times were 12.6 versus 17.6 versus 17.4 months in the early-term group, mid-term group, and the later-time group, respectively). Whereas study-2 considered 180 HCC patients treated with sorafenib in addition to MTAs in the mid- and late-term periods were divided into groups based on disease etiology (NAFLD or NASH [n = 37] and viral or alcohol [n = 143]). In study-2, there was no significant differences in overall survival between the virus/alcohol group and the NAFLD/NASH group in patients who received sequential therapy (MST was 23.4 and 27.0 months *p* = 0.173, respectively) [360]. In mice and monkeys treated with sorafenib, an equivalent of approximately one-tenth the clinical dose for HCC, effectively prevents the progression of NASH by inducing mitochondrial uncoupling and subsequent activation of AMPK [361]. In a human 3D co-culture model of NAFLD, sorafenib reduced steatosis-induced fibrogenesis [362], supporting its clinical use as a therapeutic agent for the treatment of NAFLD/NASH patients.

##### Lenvatinib

Lenvatinib was approved by FDA in 2018, and is a multiple receptor kinase inhibitor targeting VEGFA, VEGFC, VEGFR1-3, fibroblast growth factor receptor (FGFR) 1-4, stem cell factor (SCF), PDGFRα, ret proto-oncogene (RET), and kitproto-oncogene (KIT) to reduce angiogenesis and lymphoangiogenesis [15,348,350]. In a phase II study of patients with advanced HCC, 12 mg lenvatinib once daily showed a median overall survival of 18.7 months, clinical activity and had an acceptable safety profile [363]. In 2018, the phase III randomized study (REFLECT trial) displayed that lenvatinib (12 mg/day for bodyweight ≥ 60 kg or 8 mg/day for bodyweight < 60 kg) was not inferior to sorafenib (400 mg twice-daily in 28-day cycles) in overall survival (13.6 versus 12.3 months). The most common any-grade adverse events were hypertension, diarrhea, decreased appetite, and reduced weight [364]. Therefore, lenvatinib should be preferred in patients with several cardiovascular comorbidities due to the risk of severe arterial hypertension and ischemic strokes [15]. A study evaluated sixty-seven patients with unresectable advanced HCC treated with lenvatinib (26 with hepatitis C virus, 19 with hepatitis B virus, 11 with alcohol, and 11 with NASH). The PFS was significantly longer in the nonviral group than in the viral group (13.7 versus 6.6 months). Similarly, median overall survival was significantly longer in the nonviral group than in the viral group (not evaluable versus 15.9 months), thus suggesting that lenvatinib is more effective in nonviral unresectable advanced HCC [365]. In accordance, another study of lenvatinib-treated HCC patients (453 were hepatitis C virus positive, 268 hepatitis B virus positive 236 NASH correlate, and 275 had other etiologies) showed that overall survival of NASH-HCC was associated with longer median overall survival (22.2 versus 15.1 months). The PFS NASH-HCC was associated with longer median PFS (7.5 versus 6.5 months) [366]. A multi-center retrospective study also reported in patients with NAFLD/NASH (n = 103)- and Viral/Alcohol (n = 427)-related unresectable-HCC that lenvatinib treatment showed a PFS better in the NAFLD/NASH than the Viral/Alcohol group (median 9.3 versus 7.5 months) [367]. In summary, lenvatinib is effective for improving the prognosis of unresectable-HCC patients irrespective of HCC etiology.

#### 8.2.2. Second-Line Treatment for HCC

Several drugs are licensed for second-line treatment of advanced HCC by FDA including regorafenib, cabozantinib, and ramucirumab, which are multikinase inhibitors [14].

##### Regorafenib

Regorafenib was approved in 2017 as a second-line oral drug for unresectable HCC [348]. It has similar targets and structure to sorafenib, but is a more effective inhibitor of STAT3 signaling. Regorafenib inhibits kinases and cellular pathways involved in angiogenesis and tumor growth, such as the VEGFR1-3, FGFR1-2, c-KIT, PDGFRα/β, RET, and BRAF. Moreover, it inhibits various oncogenic factors such as V600-mutated BRAF, and angiopoietin-1 receptor (TIE-2) [14,348,350]. Regorafenib was approved for patients who failed sorafenib based upon the RESORCE trial, which included 374 patients with regorafenib and 193 patients with placebo. Regorafenib improved overall survival and median survival was 10.6 months for regorafenib versus 7.8 months for placebo. The most common grade 3/4 adverse events included hypertension, hand-foot skin reaction, fatigue, and diarrhea [368]. In an additional analyses from the phase III RESORCE trial, was reported a median times from the start of sorafenib to death were 26.0 months (22.6–28.1) for regorafenib and 19.2 months (16.3–22.8) for placebo [369].

##### Cabozantinib

In 2019, the FDA approved cabozantinib for patients with HCC previously treated with sorafenib. It is a multikinase inhibitor targeting VEGFR1-3, MET, the TAM family of kinases (TYRO-3, AXL, MER), RET, ROS1, KIT, TRKB, FLT-3, and TIE-2, which are mostly involved in tumor growth, angiogenesis, and immune regulation [14,348,370]. The approval in HCC was based on results from the phase III CELESTIAL trial that included 707 patients with advanced HCC who progressed on sorafenib. Median overall survival was 10.2 months with cabozantinib and 8.0 months with placebo, and median PFS survival was 5.2 months with cabozantinib and 1.9 months with placebo. The most common adverse events were hypertension, palmar-plantar erythrodysesthesia, and increased aspartate aminotransferase level, which were associated with improved outcomes [371]. A study evaluated data of an international, multicenter, real-life cohort of patients with advanced HCC treated with cabozantinib. The main liver diseases were NAFLD/NASH in 26 (30%) and hepatitis C infection in 21 (24%) patients. Cabozantinib treatment was effective and safe in patients with advanced HCC and compensated cirrhosis [372].

##### Ramucirumab

**Figure 5:**
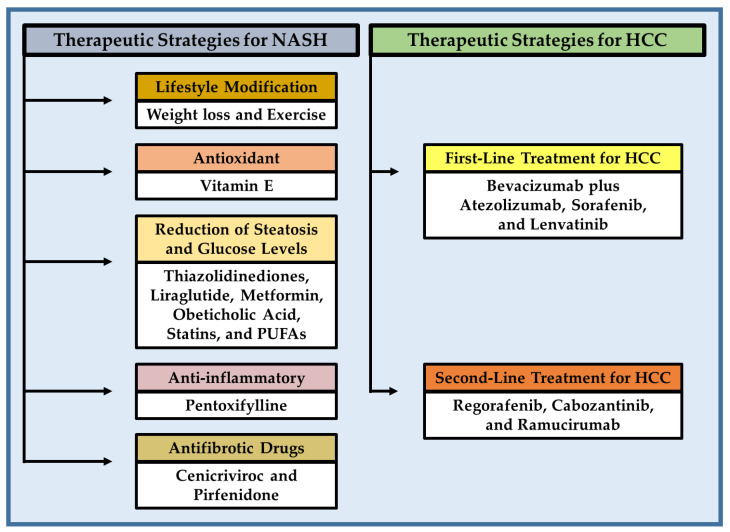
Different therapeutic strategies for NASH and HCC. Abbreviations: PUFAs, omega-3 polyunsaturated fatty acids.

Ramucirumab is an IgG-1 recombinant monoclonal antibody that inhibits VEGF-2 receptor (anti-angiogenic) and was approved in 2019 as a second-line option upon progression on sorafenib in those with elevated alpha-fetoprotein (AFP) ≥ 400 ng/mL (REACH-2 trial) [14,373]. The study reported an improved median overall survival of 8.5 versus 7.3 months in the placebo group as well as improved PFS of 2.8 versus 1.6 months. Hypertension and hyponatremia were observed as the most common adverse events [373]. The REACH trial initially reported that ramucirumab did not significantly improve survival over placebo in patients with advanced HCC [374]. Figure 5 summarizes the main therapeutic strategies reported for NASH and pharmacological treatments for HCC approved by the FDA.

## 9. Conclusions

In conclusion, NASH and HCC have a high prevalence in the world because they are closely associated with pandemic diseases such as obesity and diabetes, including metabolic syndrome and NAFLD. In the next decades these diseases will be challenge for the health care system and governments due to its high growing worldwide, especially for HCC. Although new therapies have emerged in the last decade, there not effective treatments for NASH and NASH-derived HCC, which could be related to incomplete understanding of the complexity of the mechanisms involved in the onset and progression of both NASH and HCC. To note, HCC tumors have a high heterogeneity and different genetic mutations as well as genetic disorders in the form of polymorphisms that could be determining effectiveness of current therapeutic treatments. Therefore, ongoing clinical trials should considered the most representative polymorphisms, genetic mutations, and molecular profiles to improve screening and surveillance strategies to identify biomarkers to guide personalized treatment for HCC in NASH early and delay its progression. Future directions of targeted therapy for NASH and NASH-associated HCC should be focused on several targets of pathological events driving the progression of disease and identification of biomarkers to predict treatment response of therapy. Currently, several trials are testing immune checkpoint inhibitors in combination with anti-angiogenic agents, and primary results are promising.

## Figures and Tables

**Table 1:** Genetic variants (SNPs) associated with NASH and NASH-HCC.

Pathway	Gen	Polymorphism	Effect
Lipid metabolism	*PNPLA3*	rs738409	Increase severe of NAFLD, NASH, fibrosis, and HCC [64,66,67,83,84]
	*TM6SF2*	rs10401969 (C), rs58542926 (C/T) E167K	Associated with steatosis, NASH, fibrosis/cirrhosis [69,70,82]
Metabolism of phospholipids and triacylglycerols	*LPIN1*	rs13412852 C/T	Associated with lipid levels, NASH severity, and hepatic fibrosis in children with NAFLD [87]
Lipogenesis	*HSD17B13*	rs72613567	Reduced risk of NASH and progressive liver damage [75,76]
Energy metabolism	*PPARGC1A*	rs8192678 GA/AA	Increased risk of NASH [69,70,71]
Insulin resistance	*ENPP1*	ENPP1 121Glin	Associated with fibrosis [88]
	*IRS-1*	IRS-1 972Arg	Associated with fibrosis [88]
	*GCKR*	rs780094 and rs1260326, encoding Pro446Leu	Increased serum triglycerides and associated with fibrosis [88]
Transfer of neutral lipids to nascent ApoB	*MTP*	−493 G/T	Susceptibility for NASH [74]
VLDL secretion	*APOB*	Several	Associated with NAFLD, NASH, fibrosis, and HCC [89]
	*TM6SF2*	rs58542926 C/T	Increase in NAFLD, NASH, and fibrosis [89]
De novo lipogenesis regulation	*GCKR*	rs780094 A/G	Associated with NAFLD, NASH, and fibrosis [89]
	*KLF6*	rs3750861 G/A	Decrease fibrosis [89]
Fibrosis	*AGTR1*	rs3772622	Associated with steatohepatitis and fibrosis [88]
Oxidative stress	*GCLC*	−129 C/T	Associated with NASH [88]
	*UCP2*	−866 G/A, rs695366	Reduce the risk of NASH [77,88]
Immune response	*TNF*	G238A (rs361525), G308A (rs1800629)	Susceptibility for insulin resistance, NAFLD, and NASH [88]
	*IL28B*	rs12979860 C/T	Decrease fibrosis [89]
Mitochondrial antioxidant	*SOD2*	rs4880 C/T	Increase fibrosis [89]
Phosphatidylinositol remodeling	*MBOAT7*	rs641738	Associated with NAFLD, NASH, fibrosis, and HCC [89,90]
	*HSD17B13*	rs72613567 A/T, rs143404524	Decrease NAFLD, NASH, fibrosis, and HCC [90]
Antioxidant activity	*HP*	Hp 2-2 genotype	Contribute to NASH development [73]
Repairon of the sarcolemmal membrane in skeletal muscle	*DYSF*	rs17007417	Associated with NASH-HCC [91]

Abbreviations: AGTR1, angiotensin II receptor type 1; Apo B, apolipoprotein B; ENPP1, ectoenzyme nucleotide pyrophosphate phosphodiesterase 1; GCLC, glutamate-cysteine ligase subunit catalytic; GCKR, glucokinase regulatory protein; HCC, hepatocellular carcinoma; HP, haptoglobin; HSD17B13, 17b-Hydroxysteroid dehydrogenase 13; IL-28B, interleukin 28B; IRS-1, insulin receptor substrate-1; KLF6, krueppel-like factor 6; MBOAT7, membrane-bound O-acyltransferase domain-containing 7; MTP, microsomal triglyceride transfer protein; NAFLD, non-alcoholic fatty liver disease; NASH, non-alcoholic steatohepatitis; PNPLA3, patatin-like phospholipase domain-containing protein 3; SOD2, superoxide dismutase 2; TM6SF2, transmembrane 6 superfamily member 2; TNF, tumor necrosis factor; UCP2, uncoupling protein 2; VLDL, very low-density lipoprotein.

**Table 2:** Micro RNAs dysregulated in NASH and NASH-HCC.

Micro RNAs	Stage Disease	Expression in Hepatic Tissue	Biological Function
miR-122	NAFLD, NASH, early stages of HCC	Upregulated or Downregulated	Lipid metabolism [113,114,115,127,134,138,139]
miR-29a	NASH	Downregulated	Inflammation and fibrosis [118]
miR-33	NASH	Upregulated	Lipid metabolism and transport [115]
miR-21	NASH	Upregulated	Inflammation and fibrosis [115,121]
miR-34a-5p	NASH	Upregulated	Lipid metabolism [122]
miR-122-5p	NASH	Downregulated	Lipid metabolism [122]
miR-21, miR-1290, miR-27b-3p, and miR-192-5p	NASH	Upregulated	Inflammation [115,121,130]
miR-221/222	NASH	Upregulated	Fibrosis [124]
miR99b	NASH	Upregulated	Pericellular fibrosis [126]
miR-155-5p, miR-423-5p, miR-15b-5p, miR143-3p, and miR-26a-5p	NASH	Upregulated	ATP production [130]
miR-let-7c-5p	NASH	Upregulated	Fibrogenic responses [130]
miR-423-5p, miR-let-7a-5p, and miR-let-7c-5p	NASH	Upregulated	IL-6R is decreased [130,131,133]
miR-155, miR-221/222, and miR-21,	NASH-HCC	Upregulated	Early stages of hepatocarcinogenesis [130,134,137]
miR-122	NASH-HCC	Downregulated	Early stages of hepatocarcinogenesis [134,139]
miR-16	NASH-HCC	Upregulated	Hepatocarcinogenesis [135]
miR-155, miR-193b, miR-27a, miR-31, miR-99b, miR-484, miR-574-3p, miR125a-5p, and miR-182	NAFLD-NASH-HCC	Upregulated	Hepatocarcinogenesis [134,136,137]
miR-20a, miR-200c, miR-93, miR-340-5p, and miR-720	NAFLD-NASH-HCC	Downregulated	Hepatocarcinogenesis [136]
miR-21 and miR-155	HCC	Upregulated	Early stages of HCC [134]
miR-192	NAFLD and NASH	Downregulated	HSC activation [128]
miR-34a	NAFLD and NASH	Upregulated	Inflammation [114,123,128,129]
miR-375	NAFLD and NASH	Upregulated	Glucose homeostasis, intestinal permeability modulation, and inflammation [128,129]
miR-125b	NAFLD and NASH	Upregulated	Lipid and glucose homeostasis, adipocyte differentiation, and fibrosis [128,129]
miR-33a/b	NASH	Upregulated	Lipid and cholesterol homeostasis, glucose homeostasis, and inflammation [128]
miR-451	NAFLD and NASH	Downregulated	Inflammation [128]
miR-155	NASH	Upregulated	Lipid metabolism, intestinal permeability modulation, and inflammation [125,130]

Abbreviations: ATP, adenosine triphosphate; HCC, hepatocellular carcinoma; HSCs, hepatic stellate cells; IL-6R, interleukin 6 receptor; NAFLD, non-alcoholic fatty liver disease; NASH, non-alcoholic steatohepatitis.
